# Comparative functional morphological study of the tarsal joint mobility in artiodactyls and perissodactyls in light of astragalar morphological differences

**DOI:** 10.1111/joa.70109

**Published:** 2026-01-19

**Authors:** Sei‐ichiro Takeda, Yoichi Masuda, Truong Son Nguyen, Hideki Endo

**Affiliations:** ^1^ Department of Mechanical Engineering The University of Osaka Osaka Japan; ^2^ The University Museum The University of Tokyo Tokyo Japan; ^3^ Department of Zoology, Institute of Ecology and Biological Resources, Vietnam Academy of Sciences and Technology Hanoi Vietnam; ^4^ Graduate University of Science and Technology, Vietnam Academy of Science and Technology Hanoi Vietnam

**Keywords:** cursoriality, hind limb, range of motion (ROM), three‐dimensional image analysis, ungulates

## Abstract

In most mammals, the astragalus features a single‐pulley surface for articulation with the tibia; in contrast, artiodactyls possess pulley structures at both the proximal and distal ends of the astragalus, a condition known as the double‐pulley astragalus. This structure has been hypothesized to increase the force exerted during plantarflexion by elongating the lever arm formed by the calcaneus. However, this hypothesis, based on skeletal simulations, has lacked empirical confirmation under ex vivo conditions. To address this, the present study used CT scanning to examine the hindlimbs obtained from necropsies of 15 mammalian species—including artiodactyls, perissodactyls, and a cheetah—and generated three‐dimensional (3D) reconstructions of the tarsal joint to measure mobility using the obtained 3D models. Distances between the tuber calcanei and astragalar trochleae, interosseous angles, and ranges of motion (ROMs) were quantified in maximally dorsiflexed and plantarflexed postures. In artiodactyls, the distances between the tuber calcanei and the proximal and distal trochleae of the astragalus varied with joint posture and changed strictly in antiphase, reflecting a shift in the functional locus of rotation within the astragalus. Despite these postural changes, the effective lever length relative to the instantaneous axis of rotation remained nearly constant. In species with a single‐trochlea astragalus, including perissodactyls and the cheetah, almost the entire ROM of the tarsal joint was attributable to motion at the crurotarsal joint, and the calcaneus and astragalus were virtually immobile relative to each other. By contrast, artiodactyls exhibited substantial calcaneo‐astragalar mobility, with changes in relative orientation reaching up to 90°. Tarsal joint mobility in artiodactyls was distributed between the proximal and distal astragalar joints, with ruminants showing particularly large ROMs at the distal astragalar joint. Clear differences were observed between plesiomorphic and apomorphic taxa: equids and ruminants displayed greater overall tarsal joint mobility than Tapiridae and Suina, respectively. Although Suina did not differ from ruminants in ROM at the proximal astragalar joint, they were markedly restricted in mobility at the distal astragalar joint and in calcaneo‐astragalar motion. Llamas, and likely camelids in general, exhibited tarsal joint characteristics closely resembling those of ruminants, a pattern most plausibly interpreted as convergent acquisition of similar joint configurations. These results demonstrate that the double‐pulley astragalus is associated with a distinctive pattern of joint partitioning and interosseous mobility within the tarsus, rather than with simple elongation of the calcaneal lever arm, highlighting its anatomical significance in shaping artiodactyl tarsal joint morphology.

## Graphical abstract

This study investigated how variations in the shape of the astragalus affect tarsal joint behavior, using CT scans. In artiodactyls with a double‐pulley astragalus, the calcaneus shifted plantarly during plantarflexion. This suggests the movement increases hindlimb functional length and contributes to enhanced running speed.

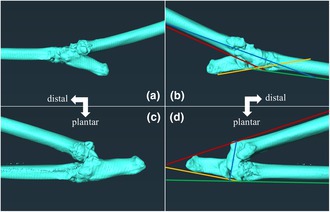

## 1 INTRODUCTION

The astragalus is one of the bones comprising the tarsal joint. The astragalus articulates proximally with the crus bones, plantarly and laterally with the calcaneus, and distally with other tarsal bones in ungulates (König & Liebich, 2007). The astragalus of various mammals is equipped with a single proximal pulley for articulation with the tibia and a distal head for the navicular (König & Liebich, 2007). The astragalus of artiodactyls, like that of other mammals, possesses the proximal pulley structure; however, it is also equipped with a distal pulley structure (König & Liebich, 2007). Thus, the artiodactyl astragalus functions as a double hinge joint between the tarsus and the tibia (Barr, 2014; Wilson & Mittermeier, 2011). This artiodactyl astragalus is called the double‐pulley astragalus (Hassanin, 2014; Prothero & Foss, 2007; Rose, 1996). The artiodactyl astragalus possesses a more deeply grooved proximal pulley than that of other mammals and articulates tightly with the crus bone (Schaeffer, 1947). Both pulleys on the artiodactyl astragalus are oriented in a sagittal direction. The groove depth and direction of these pulleys suppress inversion and eversion (i.e., pronation and supination) of the tarsal joint, restricting its movement to the sagittal direction (Schaeffer, 1947). These characteristics of the artiodactyl astragalus stabilize the tarsal joints, which are presumed to be an adaptation to cursoriality observed in early artiodactyls (Schaeffer, 1948).

The proposed advantages of the double‐pulley astragalus, which only artiodactyls possess among mammals, include limiting ankle motion in the sagittal direction and increasing the force exerted during plantarflexion (Barr, 2014; Schaeffer, 1947). Barr (2014) noted in the introduction that the functional length of the calcaneus increased by more than 10% in plantarflexion by simulation with skeletal specimens of *Ammotragus lervia*. Radiographic observations have also confirmed the displacement of the calcaneus due to dorsiflexion and plantarflexion of the tarsal joint (Schaeffer, 1947). When the distal hindlimb is viewed as leverage with the calcaneal ridge as the point of effort, the hoof as the point of load, and the astragalus as the fulcrum (axis of rotation), the functional length extension of the calcaneus corresponds to an extension of the lever arm. Lengthening the lever arm increases the force exerted by the hoof, allowing it to kick strongly on the ground. However, elongating the lever arm also results in a decrease in angular velocity. A decrease in the angular velocity of the tarsal joint affects the decrease in running speed. The limbs of artiodactyls have evolved to run faster (Gregory, 1912; Hildebrand et al., 2001; Howell, 1944; Janis & Wilhelm, 1993). If the double‐pulley astragalus lengthened the lever arm of the tarsal joint, it would imply that artiodactyl tarsal joints uniquely evolved a morphology that decreases running speed.

The purpose of this study was to clarify the morphological functions of the double‐pulley astragalus, focusing on the displacement of the calcaneus. We used necropsied specimens to simulate dorsiflexion and plantarflexion of the tarsal joint and examined calcaneal displacement under these conditions. In doing so, we aimed to reproduce ex vivo calcaneal displacement by accounting for the influence of soft tissues, which cannot be reproduced in simulations using only skeletal specimens. In this study, we used necropsied specimens of artiodactyls and perissodactyls to conduct a comparative ex vivo study of the tarsal joint biomechanics. We describe the relative changes in position of all key bones of the tarsal joint (tibia, astragalus, calcaneus, metatarsus) relative to each other in plantarflexed and dorsiflexed positions. The data obtained will allow us to characterize the mechanical advantages of the apomorphic double‐trochlea astragalus compared to the plesiomorphic single‐trochlea astragalus. We will also characterize how the ex vivo biomechanics of the tarsal joint in plesiomorphic artiodactyls (Suina) and perissodactyls (Tapiridae) differ from that of their apomorphic relatives (ruminants and equids, respectively). Finally, we will discuss the data obtained in terms of the influence of body size, habitat, and locomotor specificity on the biomechanics of the tarsal joint.

## 2 MATERIALS AND METHODS

Necropsied specimens that had been donated to The University Museum, The University of Tokyo (UMUT) were used in this study (Table 1). *Antilope cervicapra*, *Capricornis crispus*, *Cervus nippon*, *Lama glama*, *Oryx leucoryx*, *Ovis canadensis*, *Pecari tajacu*, *Potamochoerus porcus*, and *Tragelaphus spekii* are artiodactyls that possess the double‐pulley astragalus; *Equus asinus*, *Equus caballus*, *Equus grevyi*, *Tapirus indicus*, and *Tapirus terrestris* belong to perissodactyls, which are equipped with the single‐pulley astragalus; *Acinonyx jubatus* is a carnivoran with the single‐pulley astragalus (Wilson & Mittermeier, 2011). *Acinonyx jubatus* was included for comparison as cheetahs are highly cursorial, similar to artiodactyls and perissodactyls. Two individuals were used for *O. leucoryx*, *O. canadensis*, and *T. indicus*, and one individual for each of the other species. All specimens were frozen in the zoo's freezer after autopsy and subsequently transported to UMUT. After delivery, each specimen was thawed and disarticulated at the hip joint for use in the experiments. The autopsies conducted at the zoo were pathological examinations involving abdominal incisions, and no damage was observed in the hind limbs. Except for detaching the hind limb at the hip joint, no additional damage was inflicted on the specimens.

**TABLE 1:** Species used in this study.

Order	Species	Collection number	Institution	Body mass (kg)	Shape of astragalus
Artiodactyla	*Antilope cervicapra*	UMUT‐24185	Tobu zoo	32.0	Double pulley
	*Capricornis crispus*	UMUT‐22110	Kanazawa Zoological Gardens	35.1	Double pulley
	*Cervus nippon*	UMUT‐24340	Hirakawa Zoological Park	57.9	Double pulley
	*Lama glama*	UMUT‐23426	Izu Shaboten Zoo	133.0	Double pulley
	*Oryx leucoryx*	UMUT‐22132	Kanazawa Zoological Gardens	75.7	Double pulley
	*Oryx leucoryx*	UMUT‐24223	Kanazawa Zoological Gardens	79.2	Double pulley
	*Ovis canadensis*	UMUT‐22111	Kanazawa Zoological Gardens	79.3	Double pulley
	*Ovis canadensis*	UMUT‐24422	Kanazawa Zoological Gardens	78.5	Double pulley
	*Pecari tajacu*	UMUT‐23325	Izu Shaboten Zoo	21.0	Double pulley
	*Potamochoerus porcus*	UMUT‐23421	Yokohama Zoological Gardens ZOORASIA	58.8	Double pulley
	*Tragelaphus spekii*	UMUT‐24184	Tobu zoo	42.7	Double pulley
Perissodactyla	*Equus asinus*	UMUT‐24266	Yumemi Zoo	145.3	Single pulley
	*Equus caballus*	UMUT‐24285	Izu Shaboten Zoo	88.5	Single pulley
	*Equus grevyi*	UMUT‐22143	Toyama Family Park	350.0	Single pulley
	*Tapirus indicus*	UMUT‐23030	Tobu zoo	261.3	Single pulley
	*Tapirus indicus*	UMUT‐24201	Yokohama Zoological Gardens ZOORASIA	223.1	Single pulley
	*Tapirus terrestris*	UMUT‐24170	Izu Shaboten Zoo	138.5	Single pulley
Carnivora	*Acinonyx jubatus*	UMUT‐23001	Tobu zoo	38.7	Single pulley

*Note*: Species with the double‐pulley astragalus are colored gray.

**FIGURE 1:**
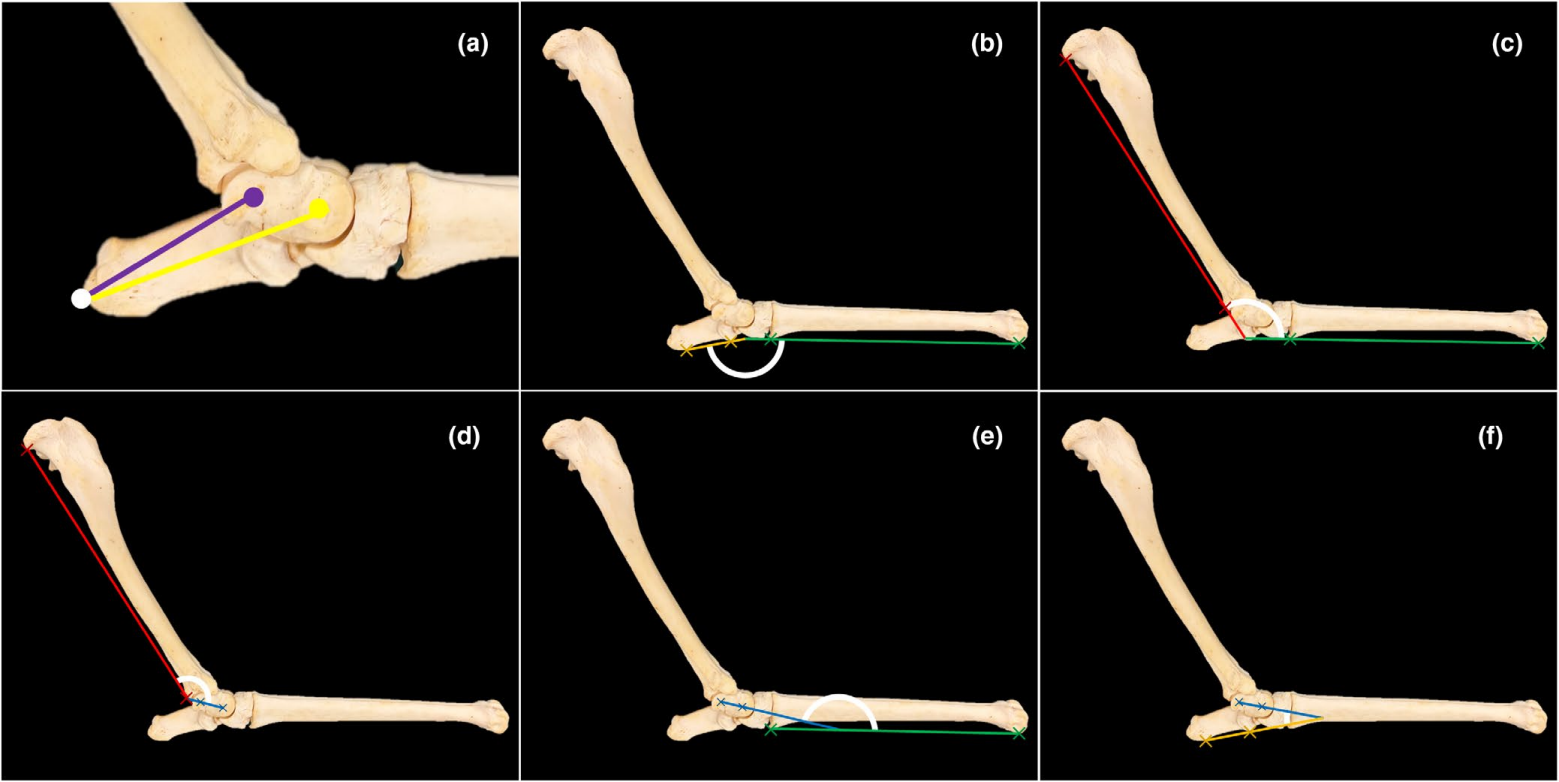
Points of measurement (left tarsal joint of *C. nippon* viewed from medial aspect). White dot: The furthest point of the tuber calcanei; purple dot: The center of the proximal astragalar trochlea; yellow dot: The center of the distal astragalar trochlea; purple line: Distance from the furthest point of the tuber calcanei to the center of the proximal astragalar trochlea; yellow line: Distance from the furthest point of the tuber calcanei to the center of the distal astragalar trochlea; red line: Line connecting the two most posteriorly projecting points of the tibia; blue line: Line connecting the two most posteriorly projecting points of the astragalus; orange line: Line connecting the two most posteriorly projecting points of the calcaneus; green line: Line connecting the two most posteriorly projecting points of the Mt3; white arc: Measured angle. (a) Lengths between the center of rotation of the tarsal joint and the furthest point of the tuber calcanei; (b) Angle between the calcaneus and Mt3; (c) Angle between the tibia and Mt3; (d) Angle between the tibia and astragalus; (e) Angle between the astragalus and Mt3; (f) Angle between the astragalus and calcaneus. The cross marks indicate the two most posteriorly projecting points of each bone.

We performed CT scanning and took measurements from the resulting 3D images. A CT scanner (Asteion PREMIUM 4 EDITION, Toshiba Medical Systems, Tokyo, Japan) was used to serially section the extremities of the limbs from the parallel distal to the proximal planes at a 0.5 mm thickness, without a gap. The current and voltage were 100 mA and 120 kV, respectively (Takeda et al., 2025). The left hindfoot was scanned in each individual. The tarsal joint was positioned to simulate dorsiflexion and plantarflexion during CT scanning. CT imaging was performed while maintaining the tarsal joint in maximally plantarflexed and dorsiflexed positions using polystyrene foam for support. The obtained series of CT images were then used to reconstruct 3D images of the hindfoot. We used these 3D images to examine the biomechanics of the tarsal joint. First, we measured the distance between the center of rotation of the tarsal joint and the furthest point of the tuber calcaneus. In species possessing a single‐pulley astragalus, one measurement was taken between the center of the proximal trochlea of the astragalus and the furthest point of the tuber calcaneus. In species with a double‐pulley astragalus, two centers of rotation are present in the astragalus; therefore, two distances were measured from a single posture of each specimen (Figure 1a). The distances measured in the plantarflexed position were subtracted from that in the dorsiflexed position, and the resulting values were normalized by body mass in kilograms to enable interspecific comparisons. Body masses referred to the mass of the specimens at the time of death (Table 1). We examined postural variation at a single measurement site and also considered the center of rotation. Specifically, in species with a double‐pulley astragalus, we calculated the difference between the distance from the center of the proximal trochlea in dorsiflexion and that from the center of the distal trochlea in plantarflexion.

Next, five angles related to the tarsal joint were measured as follows: (1) the angle between the calcaneus and third metatarsal (Mt3); (2) the angle between the tibia and Mt3; (3) the angle between the tibia and the astragalus; (4) the angle between the astragalus and Mt3; and (5) the angle between the astragalus and the calcaneus (Figure 1b–f). For the tibia, calcaneus, and Mt3, a line was drawn through the two most posteriorly projecting points. In artiodactyls, the third and fourth metatarsals are fused into a single bone. The most posteriorly projecting point of the medial trochlea of the metatarsal was used as the reference for measurement, and it is referred to as Mt3, as in other species. In the astragalus, a line passing through each rotational center was drawn in species with a double‐pulley structure. In contrast, because perissodactyls and carnivorans lack the distal rotational center, a line was drawn through the proximal rotational center and the most posteriorly projecting point of the distal articular surface. The angles formed by these lines were defined as interosseous angles. As with the distance, the ranges of motion (ROMs) between dorsiflexion and plantarflexion were calculated. Distances and angles were measured using a 3D image analyzing system (Amira 2021.1: Visage Imaging GmbH, Berlin, Germany). The centers of the astragalar trochleae were estimated by approximating the trochlear outline to a circle, using the same software as above. To examine the effects of allometry on the measured distances and angles, we performed linear regression analyses between the uncorrected measurement values and log‐transformed body mass for each astragalar structural group. Mann–Whitney U‐tests were performed on both the body mass‐adjusted length changes and the ROMs to determine significant differences between single‐pulley astragalus and double‐pulley astragalus groups. For angles involving the astragalus, no statistical comparison among groups was conducted, as the reference lines for astragalar angles differed between groups.

## 3 RESULTS

### 3.1 Length of the lever arm

**FIGURE 2:**
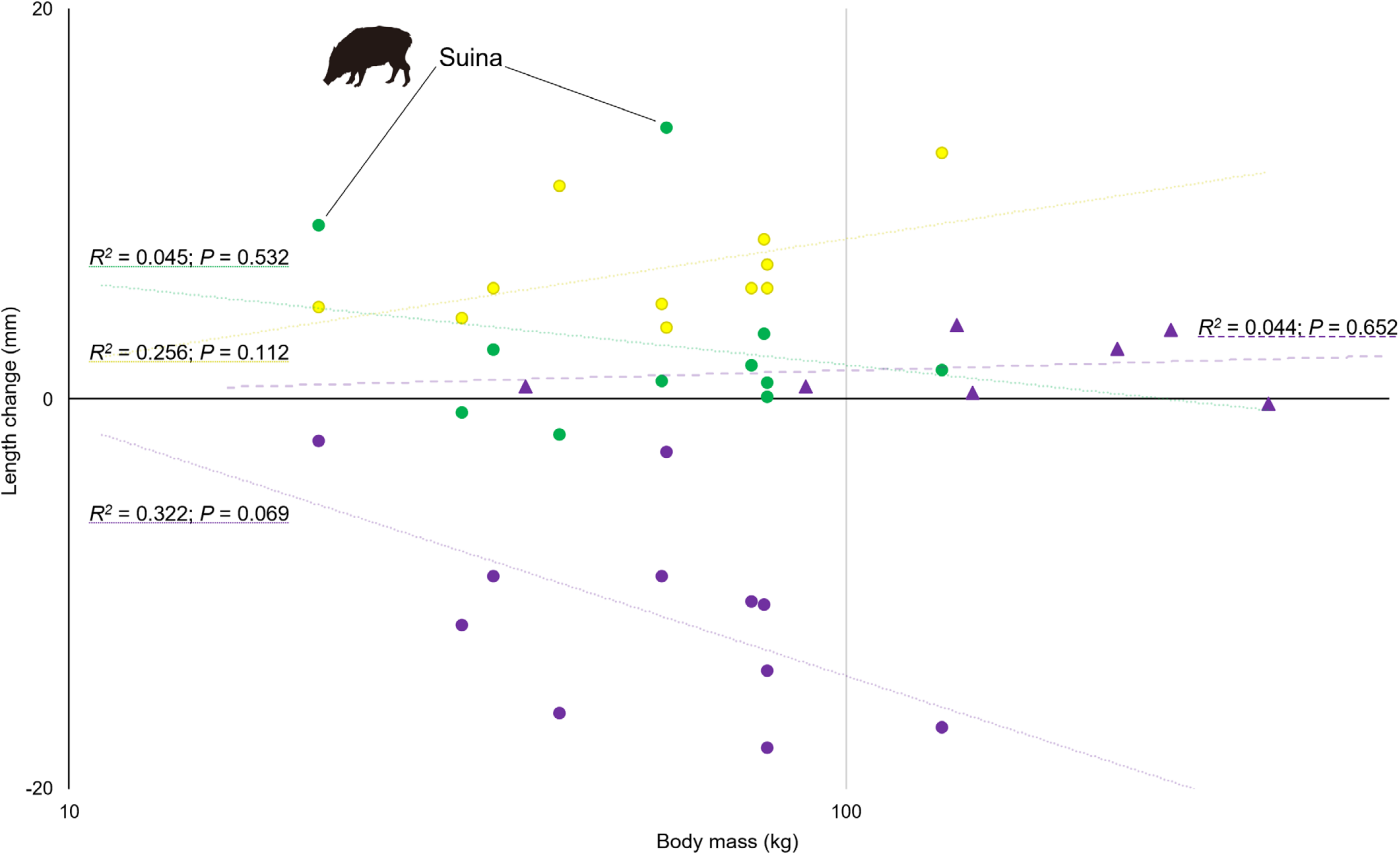
Scatter plot of length change against body mass. The horizontal axis (body mass) is on a logarithmic scale. Circles represent species with a double‐pulley astragalus, and triangles represent those with a single‐pulley astragalus. The dotted line indicates the regression line for species with a double‐pulley astragalus, and the dashed line indicates the regression line for species with a single‐pulley astragalus; the corresponding *R* ^2^ and *P* values are shown in the figure. Purple indicates the change in distance from the furthest point of the tuber calcanei to the center of the proximal astragalar trochlea; yellow indicates that to the center of the distal astragalar trochlea; green indicates the length from the distal astragalar trochlea in plantarflexion minus that from the proximal astragalar trochlea in dorsiflexion (length change between the axes of rotation).

In species with a single‐pulley astragalus, little change was observed in the distance between the astragalus and the calcaneus in dorsiflexion and plantarflexion. In contrast, in species with a double‐pulley astragalus, the distance from the center of the proximal trochlea of the astragalus increased in dorsiflexion, whereas that from the center of the distal trochlea increased in plantarflexion. However, the distance from the rotational center in each posture showed almost no change, except in two suine species (*P. tajacu* and *P. porcus*) (Figure 2; Tables 2 and 3).

**TABLE 2:** Lengths and length changes of the lever arm in the species with double‐pulley astragalus.

Species	Measurement point	State	Length (mm)	Length change between the states (mm)	Adjusted length change between the states	Length change between the axes of rotation (mm)	Adjusted length change between the axes
*A. cervicapra*	The proximal astragalus trochlea	Plantarflexion	44.6				
		Dorsiflexion	56.1	−11.6	−0.362		
	The distal astragalus trochlea	Plantarflexion	55.4				
		Dorsiflexion	51.3	4.1	0.129	−0.7	−0.023
*C. crispus*	The proximal astragalus trochlea	Plantarflexion	49.1				
		Dorsiflexion	58.1	−9.1	−0.258		
	The distal astragalus trochlea	Plantarflexion	60.7				
		Dorsiflexion	55.1	5.7	0.161	2.6	0.073
*C. nippon*	The proximal astragalus trochlea	Plantarflexion	48.6				
		Dorsiflexion	57.7	−9.1	−0.158		
	The distal astragalus trochlea	Plantarflexion	58.6				
		Dorsiflexion	53.8	4.8	0.084	0.9	0.016
*L. glama*	The proximal astragalus trochlea	Plantarflexion	64.4				
		Dorsiflexion	81.3	−16.9	−0.127		
	The distal astragalus trochlea	Plantarflexion	82.8				
		Dorsiflexion	70.2	12.6	0.095	1.5	0.011
*O. leucoryx* (UMUT‐22132)	The proximal astragalus trochlea	Plantarflexion	59.1				
		Dorsiflexion	69.5	−10.4	−0.137		
	The distal astragalus trochlea	Plantarflexion	71.2				
		Dorsiflexion	65.5	5.7	0.075	1.7	0.022
*O. leucoryx* (UMUT‐24223)	The proximal astragalus trochlea	Plantarflexion	59.0				
		Dorsiflexion	76.8	−17.9	−0.226		
	The distal astragalus trochlea	Plantarflexion	76.9				
		Dorsiflexion	70.0	6.9	0.087	0.1	0.001
*O. canadensis* (UMUT‐22111)	The proximal astragalus trochlea	Plantarflexion	61.7				
		Dorsiflexion	75.6	−13.9	−0.175		
	The distal astragalus trochlea	Plantarflexion	76.5				
		Dorsiflexion	70.8	5.6	0.071	0.8	0.011
*O. canadensis* (UMUT‐24422)	The proximal astragalus trochlea	Plantarflexion	60.9				
		Dorsiflexion	71.5	−10.6	−0.135		
	The distal astragalus trochlea	Plantarflexion	74.8				
		Dorsiflexion	66.6	8.2	0.104	3.3	0.042
*P. tajacu*	The proximal astragalus trochlea	Plantarflexion	36.4				
		Dorsiflexion	38.6	−2.1	−0.102		
	The distal astragalus trochlea	Plantarflexion	47.5				
		Dorsiflexion	42.8	4.7	0.223	8.9	0.425
*P. porcus*	The proximal astragalus trochlea	Plantarflexion	52.4				
		Dorsiflexion	55.2	−2.8	−0.047		
	The distal astragalus trochlea	Plantarflexion	69.1				
		Dorsiflexion	65.4	3.7	0.063	13.9	0.236
*T. spekii*	The proximal astragalus trochlea	Plantarflexion	51.1				
		Dorsiflexion	67.2	−16.2	−0.378		
	The distal astragalus trochlea	Plantarflexion	65.4				
		Dorsiflexion	54.5	10.9	0.255	−1.8	−0.042

*Note*: Length: distance from the measurement point to the tuber calcanei; Length change between states: dorsiflexion length minus plantarflexion length from the same measurement point; Length change between axes of rotation: length from the distal astragalus trochlea in plantarflexion minus length from the proximal astragalus trochlea in dorsiflexion; Adjusted‐: values divided by body mass (Table 1).

**TABLE 3:** Lengths and length changes of the lever arm in the species with single‐pulley astragalus.

Species	State	Length (mm)	Length change between the states (mm)	Adjusted length change between the states
*E. asinus*	Plantarflexion	61.1		
	Dorsiflexion	60.8	0.3	0.002
*E. caballus*	Plantarflexion	70.9		
	Dorsiflexion	70.2	0.7	0.008
*E. grevyi*	Plantarflexion	104.7		
	Dorsiflexion	104.9	−0.2	−0.001
*T. indicus* (UMUT‐23030)	Plantarflexion	84.3		
	Dorsiflexion	80.8	3.6	0.014
*T. indicus* (UMUT‐24201)	Plantarflexion	87.2		
	Dorsiflexion	84.6	2.6	0.012
*T. terrestris*	Plantarflexion	75.2		
	Dorsiflexion	71.3	3.8	0.028
*A. jubatus*	Plantarflexion	49.2		
	Dorsiflexion	48.5	0.7	0.018

*Note*: Length: distance from the measurement point to the tuber calcanei; Length change between states: dorsiflexion length minus plantarflexion length from the same measurement point; Adjusted‐: values divided by body mass.

In the size‐adjusted length change, the distance from the proximal trochlea of the astragalus was significantly greater in dorsiflexion in species with a double‐pulley astragalus (*p* < 0.001), whereas the difference in distance from the rotational center did not significantly differ between astragalar structural types (*p* = 0.536). In addition, regression analyses revealed no effect of body mass on any of the length changes (all *p* > 0.05; Figure 2).

### 3.2 Angles between bones in the tarsal joint

**FIGURE 3:**
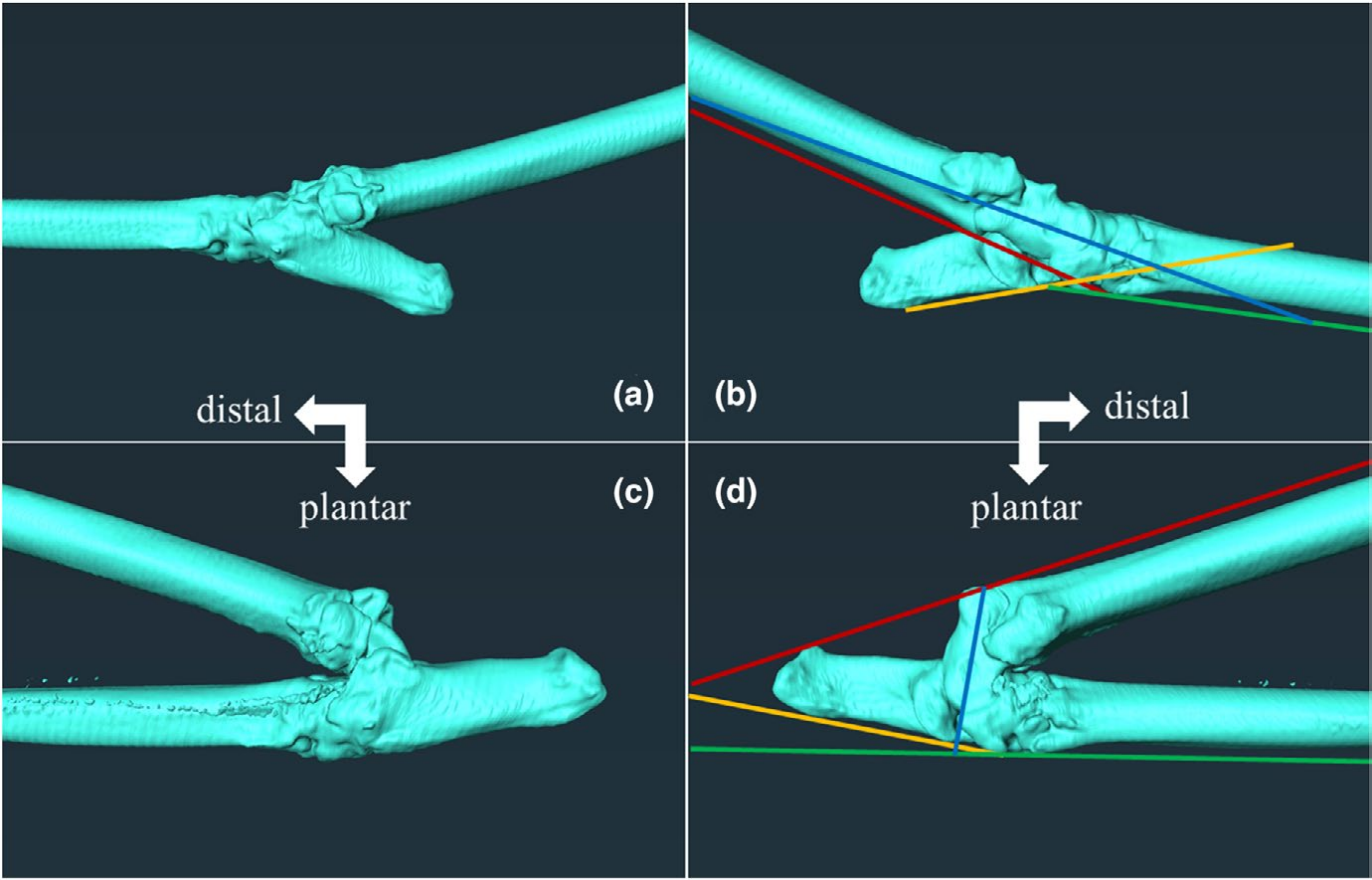
Left tarsal joint of *C. crispus*. (a) Plantarflexion viewed from lateral aspect; (b) Plantarflexion viewed from medial aspect; (c) Dorsiflexion viewed from lateral aspect; (d) Dorsiflexion viewed from medial aspect. Lines for angle measurements were added to (b) and (d). The colors of the lines are the same as in Figure 1.

**FIGURE 4:**
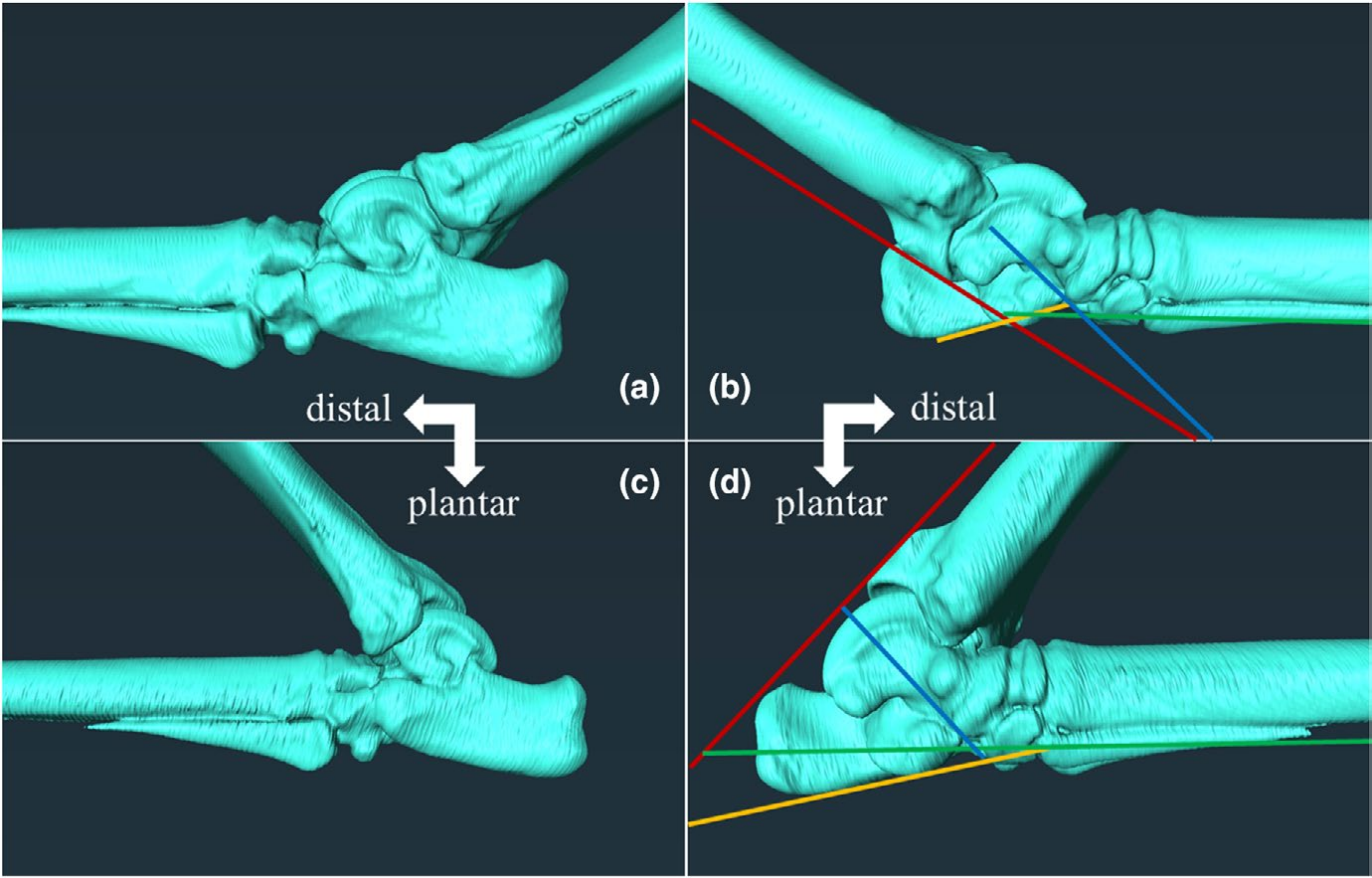
Left tarsal joint of *E. grevyi*. (a) Plantarflexion viewed from lateral aspect; (b) Plantarflexion viewed from medial aspect; (c) Dorsiflexion viewed from lateral aspect; (d) Dorsiflexion viewed from medial aspect. Lines for angle measurements were added to (b) and (d). The colors of the lines are the same as in Figure 1.

The tarsal joints of *C. crispus* (artiodactyl; double‐pulley astragalus) and *E. grevyi* (perissodactyl; single‐pulley astragalus) are shown in Figures 3 and 4, respectively. The tarsal joints of the other species are shown in Figure S1.

#### 3.2.1 Angles between calcaneus and Mt3

**FIGURE 5:**
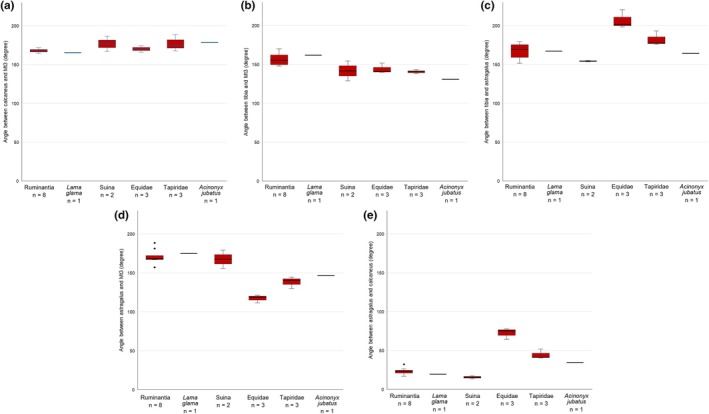
Box‐and‐whisker plots showing the interskeletal angles in plantarflexion for each taxonomic group. (a) angle between calcaneus the calcaneus and Mt3; (b) angle between the tibia and Mt3; (c) angle between the tibia and astragalus; (d) angle between the astragalus and Mt3; (e) angle between the astragalus and calcaneus.

In plantarflexed position, the tarsal joint was the most acute in *O. leucoryx* (164.5°) and the least acute in the *T. terrestris* (188.9°; Table 4). On average, the angle between calcaneus and Mt3 in plantarflexed position in artiodactyls (mean = 169.6°) was 4.1° smaller than that in perissodactyls and 9.1° smaller than that in cheetah. This angle was close in ruminants (mean = 168.3°), Suina (mean = 176.9°), equids (mean = 170.3°), and tapirs (mean = 177.2°; Figure 5a).

**TABLE 4:** Angles and ROMs.

Species	State	Angle_c‐m (°)	ROM_c‐m(°)	Angle_t‐m (°)	ROM_t‐m (°)	Angle_t‐a (°)	ROM_t‐a (°)	Angle_a‐m (°)	ROM_a‐m (°)	Angle_a‐c (°)	ROM_a‐c (°)
*A. cervicapra*	Plantarflexion	168.3		163.0		175.1		167.8		23.9	
	Dorsiflexion	187.3	19.1	24.7	138.3	116.8	58.3	92.1	75.7	84.8	60.9
*C. crispus*	Plantarflexion	172.1		161.9		173.0		168.8		19.1	
	Dorsiflexion	199.0	26.9	15.9	145.9	112.5	60.5	83.4	85.4	77.6	58.5
*C. nippon*	Plantarflexion	170.5		157.7		179.4		157.1		32.4	
	Dorsiflexion	188.8	18.3	26.9	130.8	120.2	59.2	86.7	70.4	84.5	52.1
*L. glama*	Plantarflexion	165.6		162.0		167.1		174.9		19.5	
	Dorsiflexion	186.2	20.7	2.2	159.8	104.9	62.2	77.3	97.6	83.5	64.0
*O. leucoryx* (UMUT‐22132)	Plantarflexion	166.0		148.1		156.8		188.6		22.6	
	Dorsiflexion	197.0	31.0	26.9	121.2	120.7	36.1	93.8	94.8	76.8	54.1
*O. leucoryx* (UMUT‐24223)	Plantarflexion	164.5		150.1		151.6		181.5		17.0	
	Dorsiflexion	188.3	23.8	26.1	124.0	112.9	38.6	93.1	88.4	78.6	61.6
*O. canadensis* (UMUT‐22111)	Plantarflexion	169.3		153.0		165.6		167.5		23.2	
	Dorsiflexion	189.0	19.7	35.9	117.1	128.8	36.8	92.9	74.6	83.9	60.7
*O. canadensis* (UMUT‐24422)	Plantarflexion	169.1		149.2		159.9		169.3		21.6	
	Dorsiflexion	191.2	22.1	25.5	123.7	115.3	44.6	90.2	79.2	78.6	57.0
*P. tajacu*	Plantarflexion	186.8		129.0		153.3		155.6		17.6	
	Dorsiflexion	206.0	19.3	34.5	94.4	110.5	42.9	104.1	51.5	49.9	32.2
*P. porcus*	Plantarflexion	167.1		154.6		155.4		179.3		13.7	
	Dorsiflexion	191.3	24.2	39.9	114.7	88.4	66.9	131.5	47.7	37.2	23.5
*T. spekii*	Plantarflexion	166.6		170.1		176.8		166.9		26.5	
	Dorsiflexion	188.8	22.1	5.0	165.1	126.6	50.2	58.4	108.5	112.9	86.4
*E. asinus*	Plantarflexion	166.1		140.5		201.7		118.8		75.1	
	Dorsiflexion	168.8	2.7	22.7	117.8	85.9	115.8	116.7	2.0	74.4	−0.7
*E. caballus*	Plantarflexion	170.4		152.0		220.4		111.6		78.0	
	Dorsiflexion	173.0	2.6	41.8	110.2	112.2	108.2	109.6	2.0	77.4	−0.6
*E. grevyi*	Plantarflexion	174.4		140.2		198.6		121.5		64.1	
	Dorsiflexion	176.8	2.5	39.7	100.5	113.4	85.3	106.3	15.2	76.8	12.7
*T. indicus* (UMUT‐23030)	Plantarflexion	175.1		140.6		176.1		144.5		40.4	
	Dorsiflexion	185.5	10.4	50.5	90.2	105.1	71.0	125.4	19.1	49.1	8.7
*T. indicus* (UMUT‐24201)	Plantarflexion	167.7		138.1		177.6		140.5		51.8	
	Dorsiflexion	178.3	10.6	48.3	89.8	104.5	73.1	123.8	16.8	57.9	6.2
*T. terrestris*	Plantarflexion	188.9		143.2		193.3		129.9		41.2	
	Dorsiflexion	199.7	10.8	28.3	114.9	99.1	94.2	109.2	20.8	51.1	9.9
*A. jubatus*	Plantarflexion	178.8		130.9		164.4		146.5		34.8	
	Dorsiflexion	182.5	3.8	46.6	84.3	82.3	82.1	144.3	2.2	38.3	3.5

*Note*: Species with the double‐pulley astragalus are colored gray. Angle: the angle measured from CT data at the tarsal joint during maximum dorsiflexion and plantarflexion; c‐m: between the calcaneus and third metatarsal (medial trochlea of the metatarsal in artiodactyls); t‐m: between the tibia and third metatarsal; t‐a: between the tibia and astragalus; a‐m: between the astragalus and third metatarsal; a‐c: between the astragalus and calcaneus; ROM_c‐m/a‐c: the value obtained by subtracting the angle in plantarflexion from that in dorsiflexion; ROM_t‐m/t‐a/a‐m: the value obtained by subtracting the angle in dorsiflexion from that in plantarflexion.

**FIGURE 6:**
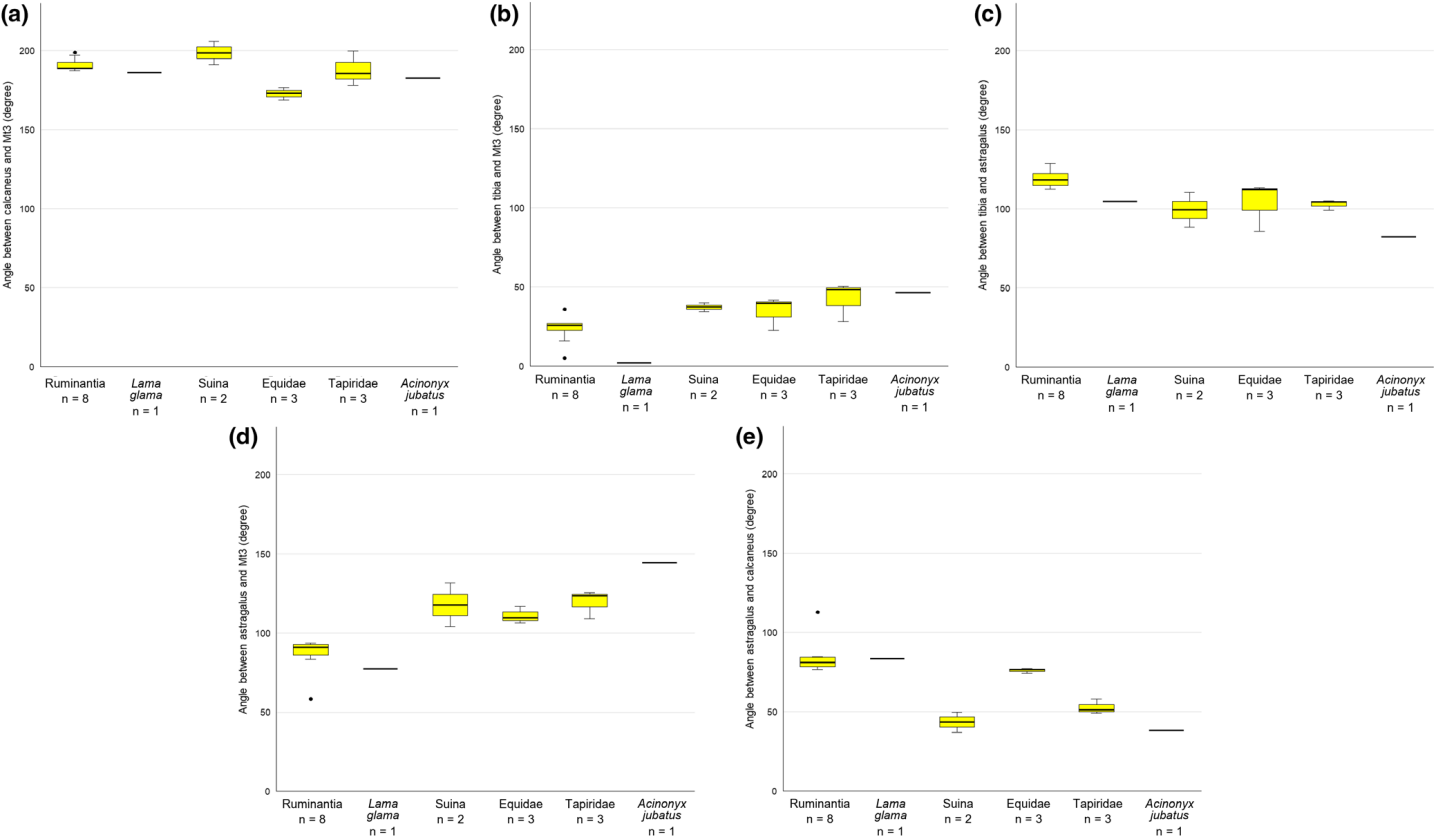
Box‐and‐whisker plots showing the interskeletal angles in dorsiflexion for each taxonomic group. (a) angle between calcaneus the calcaneus and Mt3; (b) angle between the tibia and Mt3; (c) angle between the tibia and astragalus; (d) angle between the astragalus and Mt3; (e) angle between the astragalus and calcaneus.

In dorsiflexed position the angle between calcaneus and Mt3 was the least obtuse in *E. asinus* (168.8°) and the most obtuse in *P. tajacu* (206.0°; Table 4). On average, the angle between calcaneus and Mt3 in dorsiflexed position in artiodactyls (mean = 192.1°) was 11.7° larger than that in perissodactyls and 9.6° larger than that in cheetah. This angle was also close in ruminants (mean = 191.2°), Suina (mean = 198.7°), and tapirs (mean = 187.8°). In equids (mean = 172.9°), this angle was notably less obtuse (Figure 6a).

#### 3.2.2 Angles between tibia and Mt3

In plantarflexed position, the tarsal joint was the most crouched in *P. tajacu* (129.0°) and the most straight in the *T. spekii* (170.1°; Table 4). This angle in artiodactyls was significantly larger than that in single‐pulley astragalus species (*p* < 0.01). On average, the angle between tibia and Mt3 in plantarflexed position in artiodactyls (mean = 154.4°) was 12.0° larger than that in perissodactyls and 23.5° larger than that in cheetah. This angle was very close in Suina (mean = 141.8°), tapirs (mean = 140.7°), and equids (mean = 144.2°). In ruminants (mean = 156.6°) and *L. glama* (162.0°) were markedly higher (Figure 5b).

In dorsiflexed position, the angle between tibia and Mt3 was the most acute in *L. glama* (2.2°) and the least acute in *T. indicus* (50.5°; Table 4). On average, the angle between tibia and Mt3 in dorsiflexed position in artiodactyls (mean = 24.0°) was 14.5° smaller than that in perissodactyls and 22.6° smaller than that in cheetah. This angle was also close in Suina (mean = 37.2°), tapirs (mean = 42.4°), and equids (mean = 34.7°). In ruminants (mean = 23.4°) and *L. glama*, this angle was notably more acute (Figure 6b).

#### 3.2.3 Angles between tibia and astragalus

Angles between tibia and astragalus were not directly comparable between artiodactyls and single‐pulley astragalus species because of the differences in measurements (see Methods). In artiodactyls, the angle between tibia and astragalus in plantarflexed position was the least obtuse in *O. leucoryx* (151.6°) and the most obtuse in *C. nippon* (179.4°; Table 4). On average, this angle in ruminants (mean = 167.3°) was 12.9° larger than that in Suina. Among single‐pulley astragalus species, the angle between tibia and astragalus in plantarflexed position was the least obtuse in *A. jubatus* (164.4°) and the most obtuse in *E. caballus* (220.4°; Table 4). On average, this angle in equids (mean = 206.9°) was 24.6° larger than that in tapirs (Figure 5c).

In artiodactyls, the angle between tibia and astragalus in dorsiflexed position was the most acute in *P. porcus* (88.4°) and the least acute in *O. canadensis* (128.8°; Table 4). On average, this angle in ruminants (mean = 119.2°) was 19.8° larger than that in Suina. Among single‐pulley astragalus species, the angle between tibia and astragalus in dorsiflexed position was the most acute in *A. jubatus* (82.3°) and the least acute in *E. grevyi* (113.4°; Table 4). On average, this angle in equids (mean = 103.8°) was 10.9° larger than that in tapirs (Figure 6c).

#### 3.2.4 Angles between astragalus and Mt3

Angles between astragalus and Mt3 were not directly comparable between artiodactyls and single‐pulley astragalus species because the differences in measurements (see Methods). In artiodactyls, the angle between astragalus and Mt3 in plantarflexed position was the least obtuse in *P. tajacu* (155.6°) and the most obtuse in *O. leucoryx* (188.6°; Table 4). On average, this angle in ruminants (mean = 170.9°) was 3.5° larger than that in Suina. Among single‐pulley astragalus species, the angle between astragalus and Mt3 in plantarflexed position was the least obtuse in *E. caballus* (111.6°) and the most obtuse in *A. jubatus* (146.5°; Table 4). On average, this angle in equids (mean = 117.3°) was 21.0° smaller than that in tapirs (Figure 5d).

In artiodactyls, the angle between astragalus and Mt3 in dorsiflexed position was the most acute in *T. spekii* (58.4°) and the least acute in *P. porcus* (131.5°; Table 4). On average, this angle in ruminants (mean = 86.3°) was 31.5° smaller than that in Suina. Among single‐pulley astragalus species, the angle between astragalus and Mt3 in dorsiflexed position was the most acute in *E. grevyi* (106.3°) and the least acute in *A. jubatus* (144.3°). On average, this angle in equids (mean = 110.9°) was 8.6° smaller than that in tapirs (Figure 6d).

#### 3.2.5 Angles between astragalus and calcaneus

Angles between astragalus and calcaneus were not directly comparable between artiodactyls and single‐pulley astragalus species because of the differences in measurements (see Methods). In artiodactyls, the angle between astragalus and calcaneus in plantarflexed position was the most acute in *O. leucoryx* (17.0°) and the least acute in *C. nippon* (32.4°; Table 4). On average, this angle in ruminants (mean = 23.3°) was 7.6° larger than that in Suina. Among single‐pulley astragalus species, the angle between astragalus and calcaneus in plantarflexed position was the most acute in *A. jubatus* (34.8°) and the least acute in *E. caballus* (78.0°; Table 4). On average, this angle in equids (mean = 72.4°) was 28.0° larger than that in tapirs (Figure 5e).

In artiodactyls, the angle between astragalus and calcaneus in dorsiflexed position was the least obtuse in *P. porcus* (37.2°) and the most obtuse in *T. spekii* (112.9°; Table 4). On average, this angle in ruminants (mean = 84.7°) was 41.1° larger than that in Suina. Among single‐pulley astragalus species, the angle between astragalus and calcaneus in dorsiflexed position was the least obtuse in *A. jubatus* (38.3°) and the most obtuse in *E. caballus* (77.4°; Table 4). On average, this angle in equids (mean = 76.2°) was 23.5° smaller than that in tapirs (Figure 6e).

### 3.3 ROMs in the tarsal joint

**FIGURE 7:**
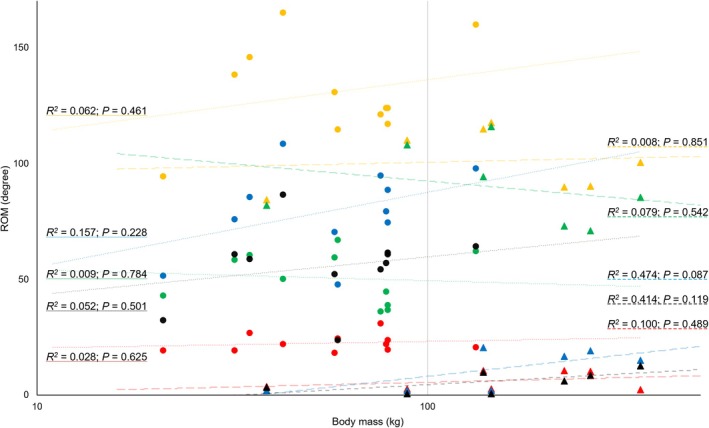
Scatter plot of angle change against body mass. The horizontal axis (body mass) is on a logarithmic scale. Circles represent species with a double‐pulley astragalus, and triangles represent those with a single‐pulley astragalus. The dotted line indicates the regression line for species with a double‐pulley astragalus, and the dashed line indicates the regression line for species with a single‐pulley astragalus; the corresponding *R* ^2^ and *P* values are shown in the figure. Red indicates the change in the angle between the calcaneus and Mt3; yellow, between the tibia and Mt3; green, between the tibia and astragalus; blue, between the astragalus and Mt3; and black, between the astragalus and calcaneus.

Regression analyses revealed no significant effect of body mass on any of the ROMs (all *p* > 0.05; Figure 7).

#### 3.3.1 ROM between calcaneus and Mt3

**FIGURE 8:**
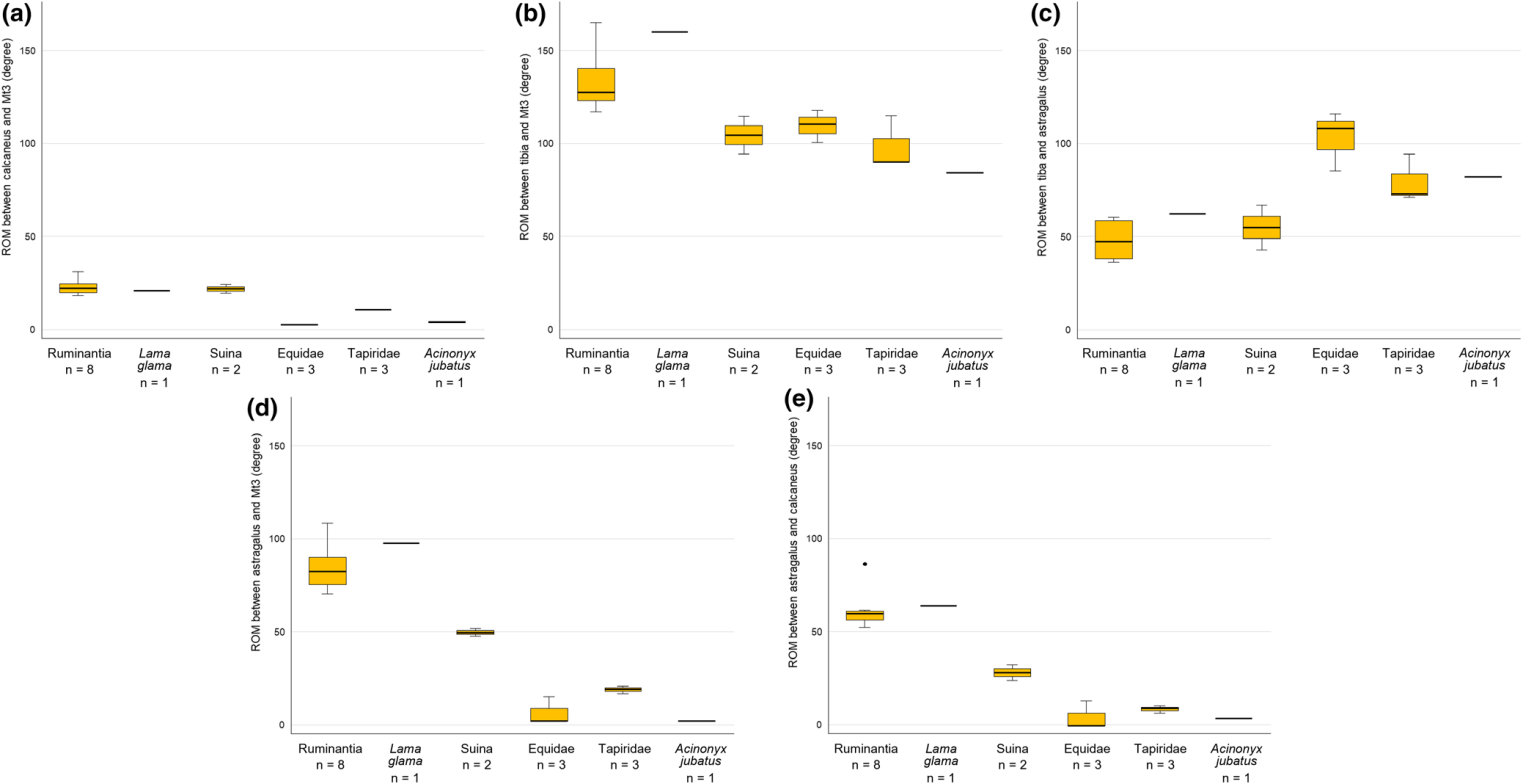
Box‐and‐whisker plots showing the range of motion (ROM) for each taxonomic group. (a) ROM between the calcaneus and Mt3; (b) ROM between the tibia and Mt3; (c) ROM between the tibia and astragalus; (d) ROM between the astragalus and Mt3; (e) ROM between the astragalus and calcaneus.

ROM between calcaneus and Mt3 varied from 2.5° in *E. grevyi* to 31.0° in *O. leucoryx* (Table 4). This ROM in artiodactyls was significantly larger than that in single‐pulley astragalus species (*p* < 0.001). On average, ROM between calcaneus and Mt3 in artiodactyls (mean = 22.5°) was 15.9° higher than that in perissodactyls and 18.7° higher than that in cheetah. Among artiodactyls, the ROM between calcaneus and Mt3 was similar in ruminants (mean = 22.8°), *L. glama* (20.7°), and Suina (mean = 21.7) on average. In perissodactyls, this ROM in equids (mean = 2.6°) was 8.0° smaller than that in tapirs (Figure 8a).

#### 3.3.2 ROM between tibia and Mt3

ROM in the tarsal joint varied from 84.3° in *A. jubatus* to 165.1° in *T. spekii* (Table 4). This ROM in artiodactyls was significantly larger than that in single‐pulley astragalus species (*p* < 0.01). On average, mobility in the tarsal joint in artiodactyls (mean = 130.5) was 26.6° higher than that in perissodactyls and 46.2° higher than that in the cheetah. At the same time, tarsal joint mobility in ruminants (mean = 133.3°) was markedly higher than that in Suina (mean = 104.6°). Tarsal joint mobility in llamas was high (159.8°) and exceeded the average for ruminants. Among perissodactyls, the ROM in the tarsal joint in equids (mean = 109.5°) was only slightly higher than that in Suina and, on average, 11.2 degrees higher than in tapirs (Figure 8b).

#### 3.3.3 ROM between tibia and astragalus

In artiodactyls, ROM between the tibia and astragalus varied from 36.1° in *O. leucoryx* to 66.9° in *T. porcus* (Table 4). On average, ROM between the tibia and astragalus in ruminants (mean = 48.0°) was 6.9° smaller than that in Suina. Among single‐pulley astragalus species, ROM between the tibia and astragalus varied from 71.0° in *T. indicus* to 115.8° in *E. asinus* (Table 4). On average, ROM between the tibia and astragalus in equids (mean = 103.1°) was 23.7° smaller than that in tapirs (Figure 8c).

#### 3.3.4 ROM between astragalus and Mt3

In artiodactyls, ROM between the astragalus and Mt3 varied from 47.7° in *T. porcus* to 108.5° in *T. spekii* (Table 4). On average, ROM between astragalus and Mt3 in ruminants (mean = 84.6°) was 35.0° larger than that in Suina. Among single‐pulley astragalus species, ROM between astragalus and Mt3 varied from 2.0° in *E. asinus* and *E. caballus* to 20.8° in *T. terrestris* (Table 4). On average, ROM between astragalus and Mt3 in equids (mean = 6.4°) was 12.5° smaller than that in tapirs (Figure 8d).

#### 3.3.5 ROM between astragalus and calcaneus

In artiodactyls, ROM between the astragalus and calcaneus varied from 23.5° in *T. porcus* to 86.4° in *T. spekii* (Table 4). On average, ROM between astragalus and calcaneus in ruminants (mean = 61.4°) was 33.5° larger than that in Suina. Among single‐pulley astragalus species, ROM between astragalus and calcaneus varied from −0.7° in *E. asinus* to 12.7° in *E. grevyi* (Table 4). On average, ROM between astragalus and calcaneus in equids (mean = 3.8°) was 4.5° smaller than that in tapirs (Figure 8e).

## 4 DISCUSSION

In ungulates, dorsiflexion of the tarsal joint reaches its maximum during the middle of the swing phase, whereas plantarflexion is greatest at the transition from the stance phase to the swing phase (Back et al., 1995; Gambaryan, 1974). In vivo ROM in the tarsal joint reported for running ungulates is 44–85° (Alexander et al., 1982; Back et al., 1995; Deuel & Lawrence, 1987; Kobluk et al., 1989; Orgebin et al., 2025). In this study, the change in angle between the tibia and Mt3 ranged from 84.3° to 165.1° (Figure 8b; Table 4). Two factors may account for this discrepancy. The first factor is the difference in measurement methods. In vivo data were obtained by placing markers on the limb joints and measuring the angle formed by lines connecting the markers. If the marker‐based lines did not coincide with the anatomical axes defined by the skeletal morphology, the measured angles would be expected to deviate from those obtained in this study. However, in horses, the maximum dorsiflexion angle recorded in vivo differed little from the plantarflexion angle obtained in this study (Deuel & Lawrence, 1987; Kobluk et al., 1989), whereas the dorsiflexion angle was substantially greater in the in vivo data. This suggests that the influence of measurement methodology is not substantial. The second possible explanation is that the tarsal joint during locomotion may not dorsiflex to its full anatomical potential. This is supported by the tarsal angles observed in horses. During the swing phase, the limb is flexed and subsequently protracted forward before being extended (Back et al., 1995; Gambaryan, 1974). For forward protraction of the limb, maximal joint flexion is not required provided that the distal limb does not contact the ground. Therefore, the difference in ROM between in vivo and skeletal data likely reflects the limited functional use of the joint during running. The larger ROM observed in the skeletal data may be utilized during locomotor activities other than running, as well as during non‐locomotor behaviors such as sitting while flexing the limbs on the ground.

The morphology of the astragalus and calcaneus is strongly influenced by body size (Barr, 2014, 2020; Curran, 2012; Gruwier & Kovarovic, 2022; Orgebin et al., 2025). Accordingly, the ROM among the constituent bones of the tarsal joint might also be expected to vary with body size. However, no significant size effect was detected for any of the joint movements examined in this study. Since body size alters the mechanical loads acting on the bones, skeletal features such as bone robustness and the shape of muscle attachment sites often vary accordingly (Martinez & Sudre, 1995; Tsubamoto, 2014; Wimberly, 2023). Nevertheless, the ROM of the tarsal joint may not serve as a major functional adaptation to such loading. Large mammals tend to employ more vertical limbs than smaller ones, thereby supporting body weight more efficiently during locomotion (Biewener, 1990, 2005). In vivo data indicate that in large mammals during locomotion, the angles in the limb joints (including the tarsal joint) remain sufficiently straightened during the stance phase (Gambaryan, 1974). Examination of tarsal joint mobility in even larger taxa—such as cattle, hippopotamuses, and rhinoceroses—may reveal whether body size exerts a more pronounced effect on joint kinematics.

Barr (2014) noted in the introduction that the double‐pulley astragalus extends the lever arm by more than 10% of its length from dorsiflexion to plantarflexion. Indeed, in species possessing a double‐pulley astragalus, the distance from the rotational axis of the astragalus to the tuber calcanei varied with joint posture. However, when focusing on the rotational locus of the tarsal joint itself, the distance from the center of astragalar rotation to the tuber calcanei remained nearly constant across all examined species. This finding suggests that the double‐pulley structure of the astragalus functions to maintain a constant lever arm length, even when the rotational axis shifts during joint movement. The length of the lever arm did not increase in species with double‐pulley astragalus any more than in those with a single‐pulley astragalus, indicating that the double‐pulley astragalus does not function to enable a powerful kick on the ground at the expense of speed.

**FIGURE 9:**
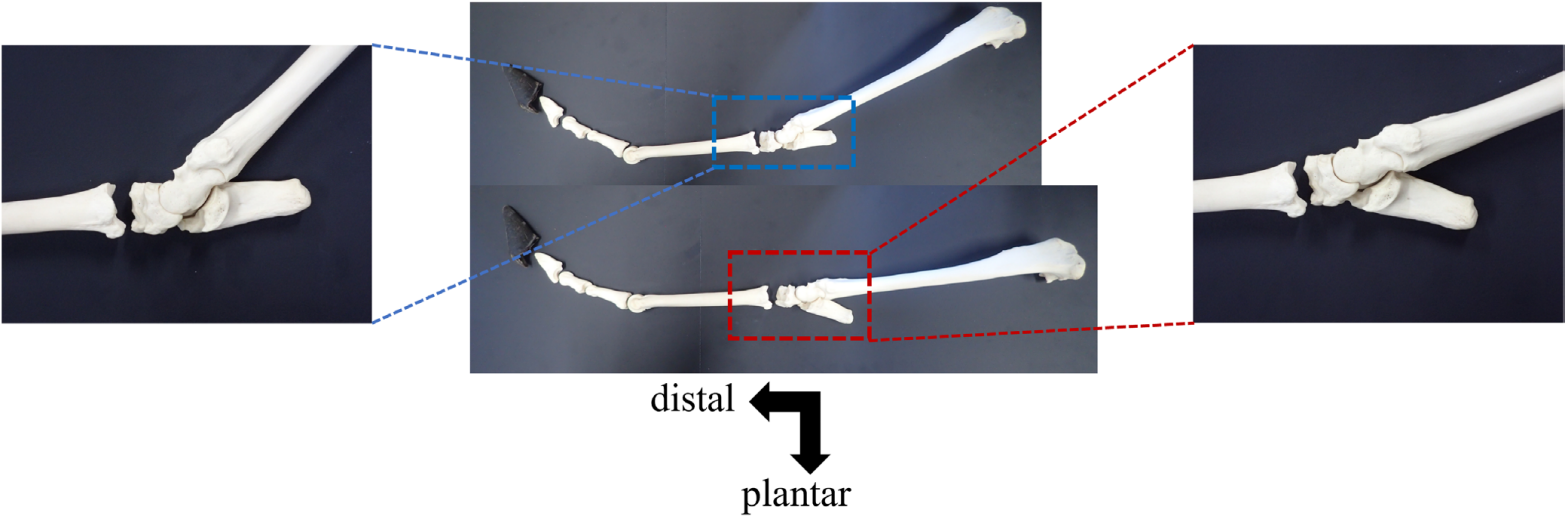
Simulation of functional length in plantarflexion by skeletal specimen (right hindlimb of *C. crispus* viewed from medial aspect). Left: Tarsal joint when no displacement of the calcaneus. Top center: Skeletal arrangement distal to the tibia when no displacement of the calcaneus. Bottom center: Skeletal arrangement distal to the tibia when the calcaneus moves plantarly. Right: Tarsal joint when the calcaneus moves plantarly.

The angle changes, however, were clearly different between species with a single‐pulley astragalus and those with a double‐pulley astragalus. In species with a single‐pulley astragalus, the ROM between the calcaneus and Mt3 ranged from 2 to 10 degrees. In contrast, in species with double‐pulley astragalus, the ROM between the calcaneus and Mt3 exceeded 19 degrees in all nine species used in this study (Figure 8a; Table 4). In addition, the angles between the tibia and Mt3—that is, the overall ROM of the tarsal joint—were significantly greater in species possessing a double‐pulley astragalus (Figure 8b; Table 4). Species possessing a double‐pulley astragalus also exhibited a greater maximum plantarflexion angle of the tarsal joint than those with a single‐pulley astragalus (Figure 5b; Table 4). These suggest that the function of the double‐pulley astragalus is to move the calcaneus to the plantar side during plantarflexion. As skeletal specimens that were not attached by ligaments or muscles for simulation were used (Barr, 2014), it is anticipated that it would have been difficult to determine the precise direction of calcaneal movement. An advantage of the calcaneus tilting toward the plantar side during plantarflexion may be the extension of the stride. When the calcaneus remains in a fixed position, the tibia can make contact with the calcaneus during plantarflexion, which prevents the tarsal joint from extending straight. By moving the calcaneus to the plantar side during plantarflexion, the tibia is not hindered by the calcaneus, allowing the angle of the tarsal joint to approach 180 degrees. A straight extension of the tarsal joints increases the functional length of the hindlimb. This functional lengthening of the hindlimb corresponds to an increase in stride length during running (Figure 9). As the stride lengthens, the distance traveled per step increases, and thus the running speed increases. This suggests that the tarsal joint with a double‐pulley astragalus may be a mechanism that enhances running speed. However, in this study, only the conditions of maximum plantarflexion and maximum dorsiflexion were examined. Recording intermediate joint positions is expected to reveal additional functional advantages conferred by the double‐pulley astragalus.

The magnitude of angle change varied among species with a double‐pulley astragalus. This predicts that the angle change of the calcaneus is adaptationally influenced by the morphological and ecological characteristics of the species. Indeed, mobility of leg joints depends on habitat and joint structure (Endo et al., 2019; Takeda et al., 2023, 2025). In addition, the shape of the astragalus of bovids differs depending on the habitat (Barr, 2014; DeGusta & Vrba, 2003; Plummer et al., 2008; Weinand, 2007). In bovids, plains species possess relatively small astragali, whereas forest‐dwelling species exhibit relatively large astragali (Barr, 2014). This difference was explained by the extension of the lever arm due to calcaneal displacement. Plains species increase the speed of tarsal joint rotation by not extending the length of the lever arm, whereas forest species increase the muscle force during plantarflexion by extending the length of the lever arm (Barr, 2014). However, the calcaneus was found to be displaced plantarly instead of proximally in this study. If the distance of calcaneus displacement varies with the size of the astragalus, it might be expected that the astragalus of species that inhabit complex terrain, such as forests, would be larger than that of plains species. For example, among the artiodactyls used in this study, *A. cervicapra* and *O. leucoryx* are plains species, and *O. canadensis* lives in mountainous areas (Wilson & Mittermeier, 2011; Table 1). In this study, *A. cervicapra* and *O. leucoryx* were expected to show less angle change than *C. crispus* and *O. canadensis*. Indeed, *A. cervicapra* showed the least change in the angle between the calcaneus and Mt3 among species with a double‐pulley astragalus. However, the angle changes of *O. leucoryx* were larger than those of *O. canadensis* (Table 4). The results of this study showed no clear trend between the angle change of the calcaneus and habitat. In contrast, among species possessing a double‐pulley astragalus, Suina (*P. tajacu* and *P. porcus*) exhibited a smaller ROM in the tarsal joint, and the distal pulley of the astragalus showed limited rotation (Figure 8b; Table 4). Suina have relatively shorter limbs compared with other artiodactyls (Wilson & Mittermeier, 2011). Consequently, the advantage gained from increasing stride length is smaller than in other groups. This suggests that limb length may influence the ROM of the tarsal joint. In addition, Suina are sometimes regarded as models of Paleogene artiodactyls based on characteristics such as digit number, the lack of fusion of the metatarsals, and limb bone proportions (Clifford, 2010). If these features of Suina are considered representative of early artiodactyls, this implies that during artiodactyl evolution, increased rotation at the joint articulating with the distal pulley of the astragalus occurred after the acquisition of a double‐pulley astragalus. In other words, the acquisition of a double‐pulley astragalus and the expansion of the ROM at the distal pulley of the astragalus may not have occurred simultaneously. From this perspective, it is suggested that additional functional advantages of the double‐pulley astragalus remain to be discovered. In contrast, camelids diverged earlier than Suina (dos Reis et al., 2012; Song et al., 2012; Upham et al., 2019); however, the ranges of motion of the tarsal joints in the llama (a camelid) were more similar to those of ruminants than to those of Suina (Figure 8; Table 4). Therefore, the mechanical properties of the tarsal joint in camelids are unlikely to have been inherited from the common ancestor of artiodactyls and are instead inferred to have evolved convergently with those of ruminants.

Among artiodactyls, running forms can be broadly categorized into cursorial forms, which increase running speed by elevating stride frequency, and saltatorial forms, which achieve higher speed by lengthening stride length (Gambaryan, 1974). The mechanical advantage produced by the inclination of the calcaneus through astragalar rotation is likely to be more pronounced in saltatorial species. In this study, *O. leucoryx* was classified as a cursorial form and *C. crispus* as a saltatorial form (Belyaev et al., 2022; Gambaryan, 1974). Although the inclination of the calcaneus in *C. crispus* fell between the two *O. leucoryx* individuals, *C. crispus* exhibited a larger ROM in the tarsal joint and a greater angular displacement between the tibia and astragalus (Table 4). These factors are predicted to affect variation in joint mobility. It is expected that increasing the number of species for which calcaneal angle changes could be examined will clarify the relationship between the angle of calcaneal change and body size, habitat, and phylogenetic relationships.

Like Suina, tapirs retain plesiomorphic features in their limbs that are similar to those of Paleogene ancestral taxa (Holbrook, 2001, 2009; Janis, 1984). Although the rotation of the distal articular surface of the astragalus in tapirs was less pronounced than that in artiodactyls, it was greater than that in equids (Figure 8; Table 4), suggesting that equids have evolved toward restricting movement of the distal articular surface of the astragalus. Limb joints in ungulates are generally structured to limit movement to the direction of progression (Cope, 1889; Hildebrand et al., 2001; Shotwell, 1961). It is therefore inferred that equids evolved by stabilizing the distal articular surface of the astragalus to prevent movements outside the direction of progression. However, tapir limb morphology also exhibits features that differ from those of ancestral taxa (MacLaren & Nauwelaerts, 2017, 2020); therefore, rotation of the distal articular surface of the astragalus in perissodactyls may not represent a plesiomorphic condition but instead a derived trait acquired by tapirs.

In horses, which possess a single‐pulley astragalus, the ridges of the astragalus pulleys run obliquely in a medial to lateral direction, causing the hooves to move anteriorly and laterally during dorsiflexion (König & Liebich, 2007) The morphology of the astragalus also affects the talocrural joint in horses: the joint rotates with a slight torsion, and during the swing phase, the tarsal joint abducts by approximately 10° compared with the stance phase (Badoux, 1987; Lanovaz et al., 2002). The orientation of the astragalus pulleys allow the tibia to extend unimpeded by the calcaneus during plantarflexion in horses with a single‐pulley astragalus. However, since the axis of rotation of the tarsal joint is tilted relative to the sagittal direction, energy is wasted in outward motion of the tarsal joint during running. In contrast, the ridges of the astragalus pulleys of ruminants are oriented sagittally (König & Liebich, 2007). The double‐pulley astragalus enables artiodactyls to extend and flex the tarsal joints only in the sagittal direction. Since the axis of rotation of the tarsal joint is oriented perpendicular to the sagittal direction, the motion of the tarsal joints is efficiently converted into forward speed when running. This suggests that the tarsal joints of artiodactyls may be more adapted for cursoriality than those of horses.

## 5 CONCLUSION

This study demonstrates that the double‐pulley astragalus in artiodactyls does not function to increase the effective lever arm length of the calcaneus during plantarflexion. When shifts in the rotational center of the astragalus are considered, the distance between the functional axis of rotation and the tuber calcanei remains nearly constant across joint postures and astragalar types. Thus, the double‐pulley astragalus does not enhance propulsive force at the expense of angular velocity.

Instead, species possessing a double‐pulley astragalus exhibit markedly greater calcaneal displacement and a larger range of motion in the tarsal joint, particularly in dorsiflexion. This increased mobility allows the calcaneus to shift plantarly during plantarflexion, preventing mechanical interference with the tibia and enabling near‐complete extension of the tarsal joint. As a result, the functional length of the hindlimb may be increased, leading to a longer stride without sacrificing sagittal‐plane efficiency.

These findings indicate that the primary functional advantage of the double‐pulley astragalus lies in facilitating stride elongation through enhanced joint excursion rather than through lever arm elongation. Variation among artiodactyl clades further suggests that the degree to which this mechanism is exploited has evolved in association with limb proportions and locomotor strategies.

## AUTHOR CONTRIBUTIONS

**Sei‐ichiro Takeda:** Conceptualization; data curation; formal analysis; investigation; methodology; project administration; validation; visualization; writing—original draft; writing—review and editing. **Yoichi Masuda:** Funding acquisition; writing—review and editing. **Truong Son Nguyen:** Formal analysis; investigation. **Hideki Endo:** Funding acquisition; resources; writing—review and editing.

## Supporting information


**Figure S1.** Left tarsal joints of 18 individuals. (a) dorsiflexion viewed from lateral aspect. (b) dorsiflexion viewed from medial aspect. (c) plantarflexion viewed from lateral aspect. (d) plantarflexion viewed from medial aspect.

## Data Availability

The data that support the findings of this study are available from the corresponding author upon reasonable request.
