# Supplementary material for: Comparative functional morphological study of the tarsal joint mobility in artiodactyls and perissodactyls in light of astragalar morphological differences
Source: J Anat. 2026 Jan 19;249(3):587–603. doi: 10.1111/joa.70109 (PMC13399001; doi:10.1111/joa.70109)
Supplement: Supplementary file 1 — Figure S1. Left tarsal joints of 18 individuals. (a) dorsiflexion viewed from lateral aspect. (b) dorsiflexion viewed from medial aspect. (c) plantarflexion viewed from lateral aspect. (d) plantarflexion viewed from medial aspect. [file JOA-249-587-s001.pdf]

Figure S1. Left tarsal joints of 18 individuals. A) dorsiflexion viewed from lateral aspect. B) dorsiflexion viewed from medial aspect. C) plantarflexion viewed from lateral aspect. D) plantarflexion viewed from medial aspect.

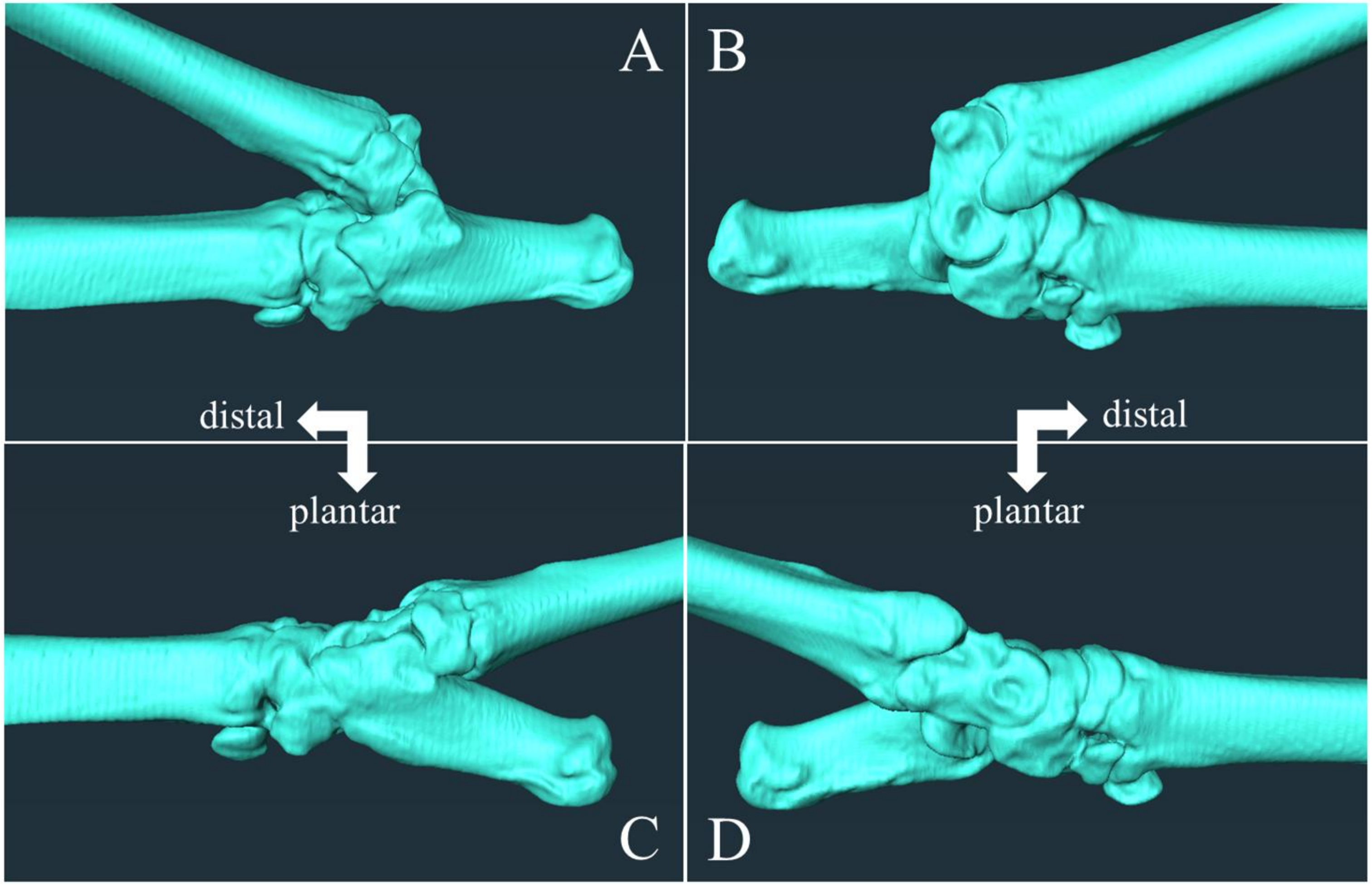

Antilope cervicapra

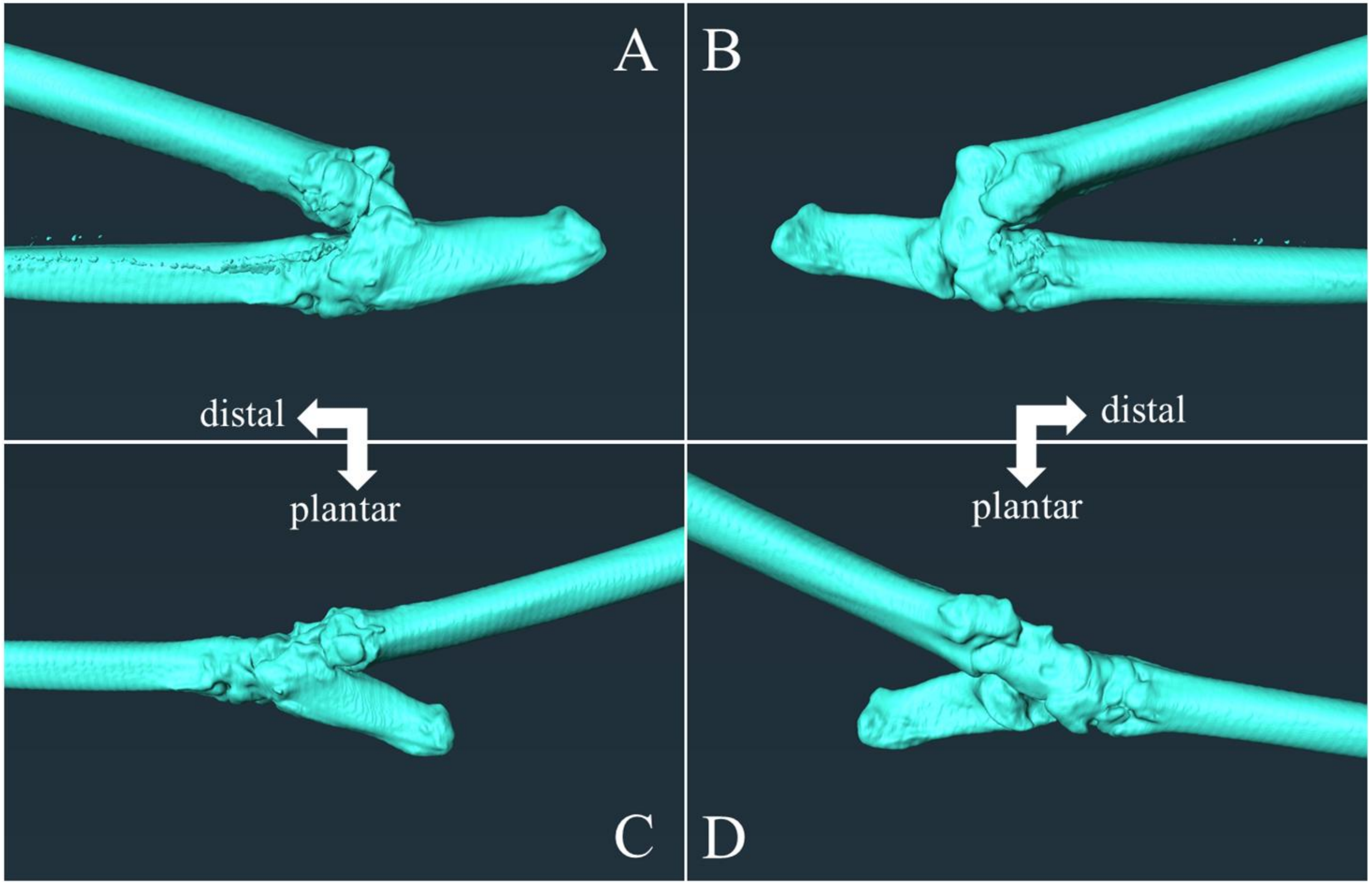

Capricornis crispus

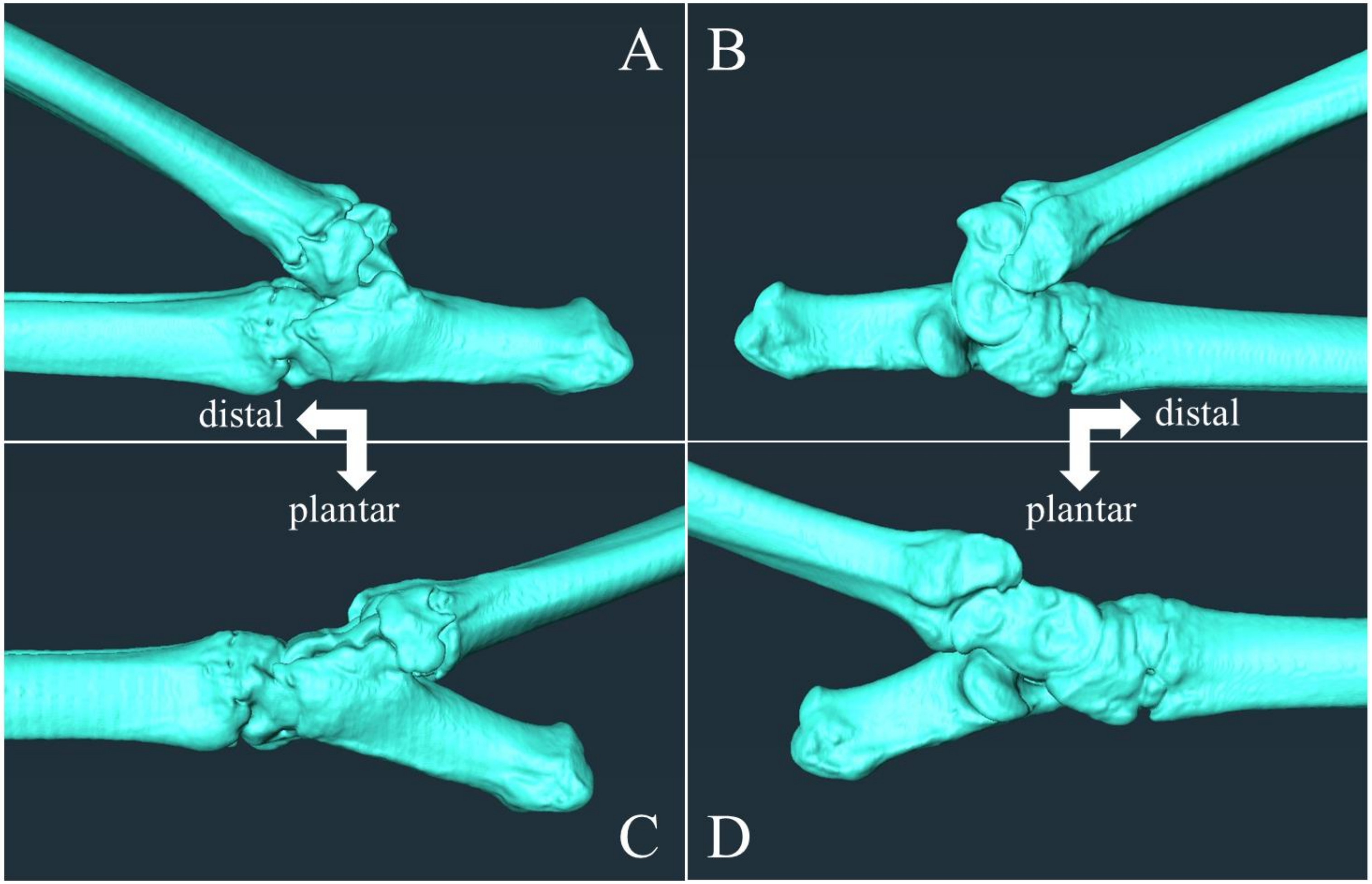

Cervus nippon

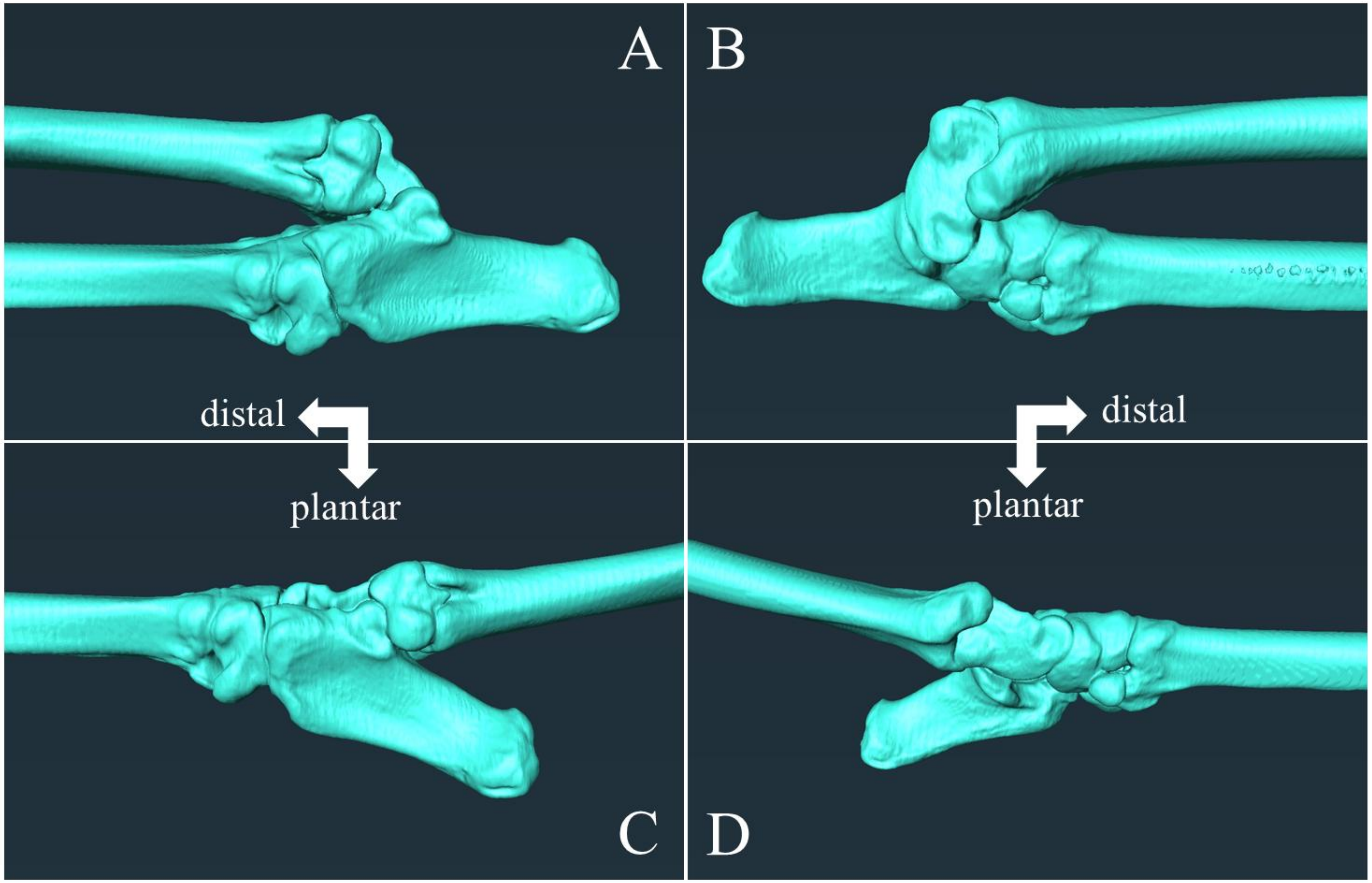

Lama glama

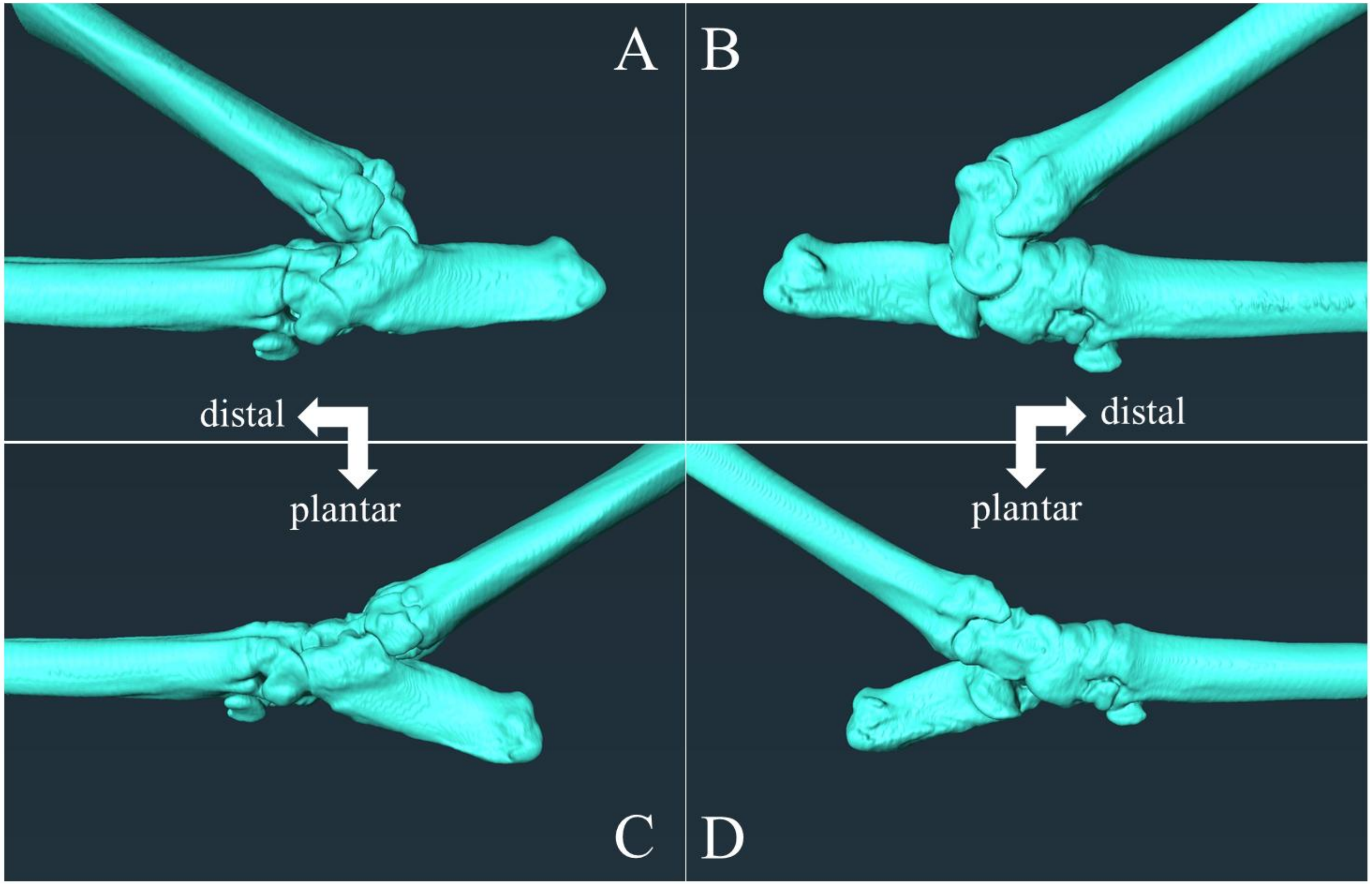

Oryx leucoryx (UMUT-22132)

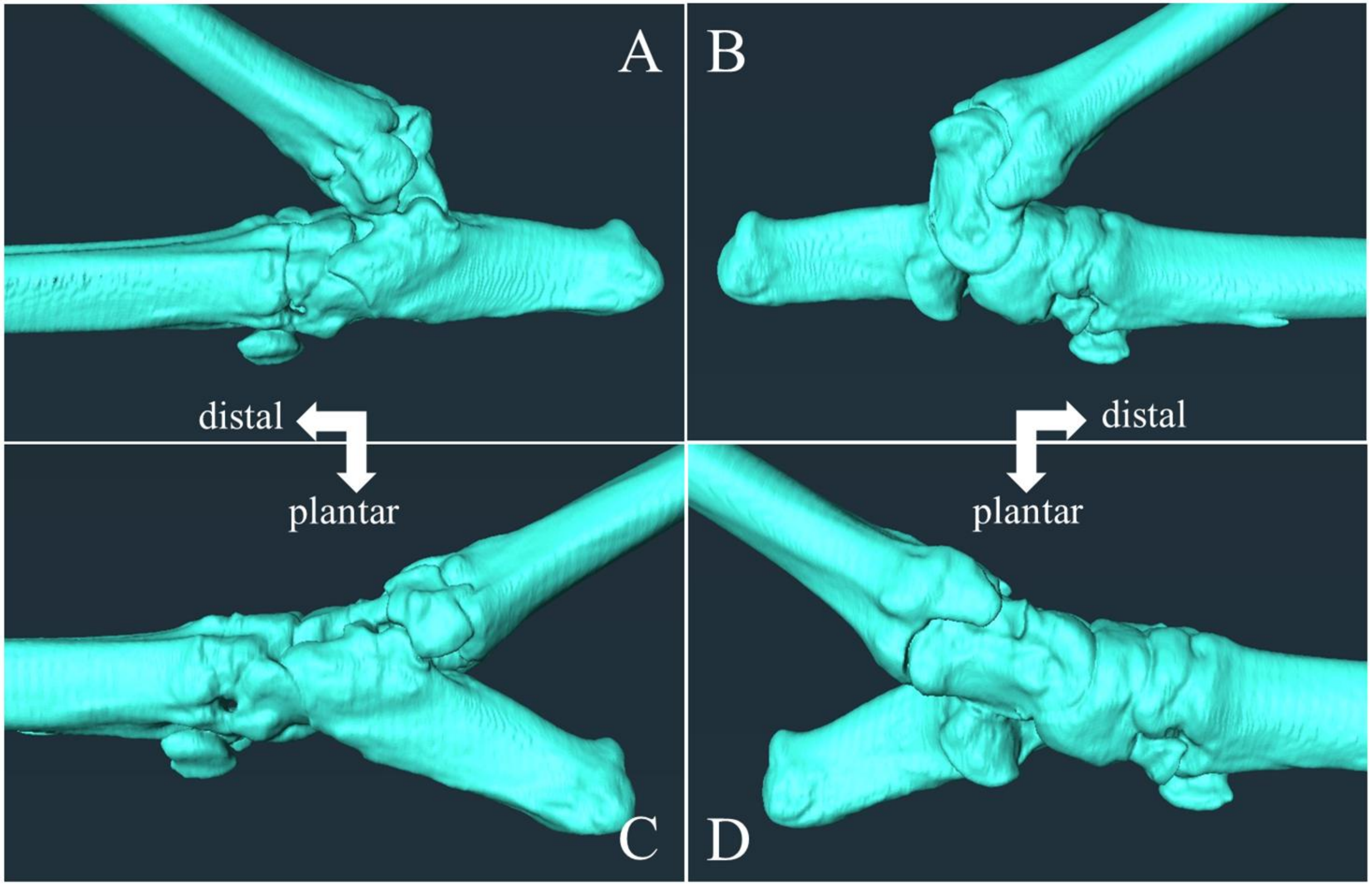

Oryx leucoryx (UMUT-24223)

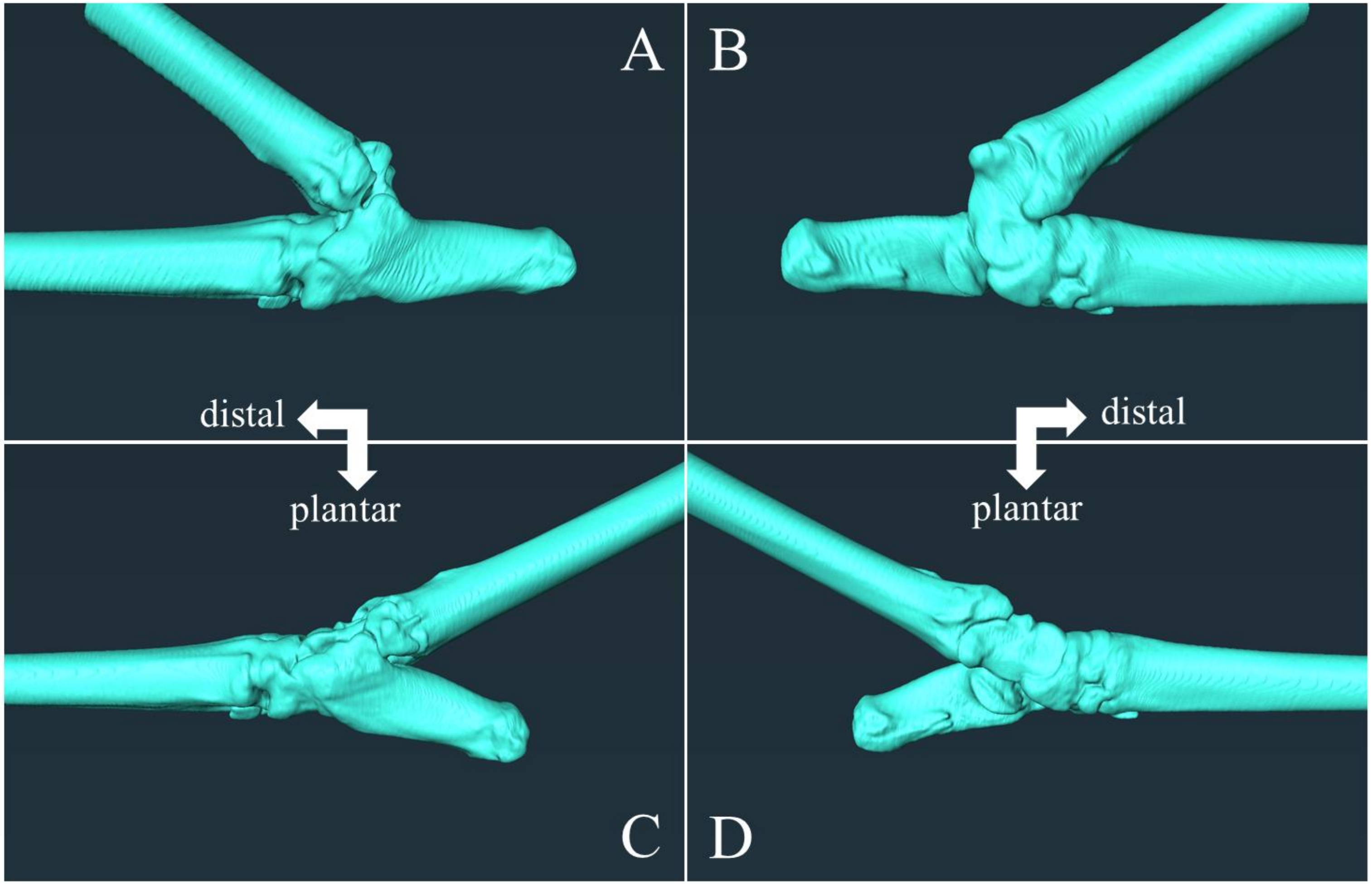

Ovis canadensis (UMUT-22111)

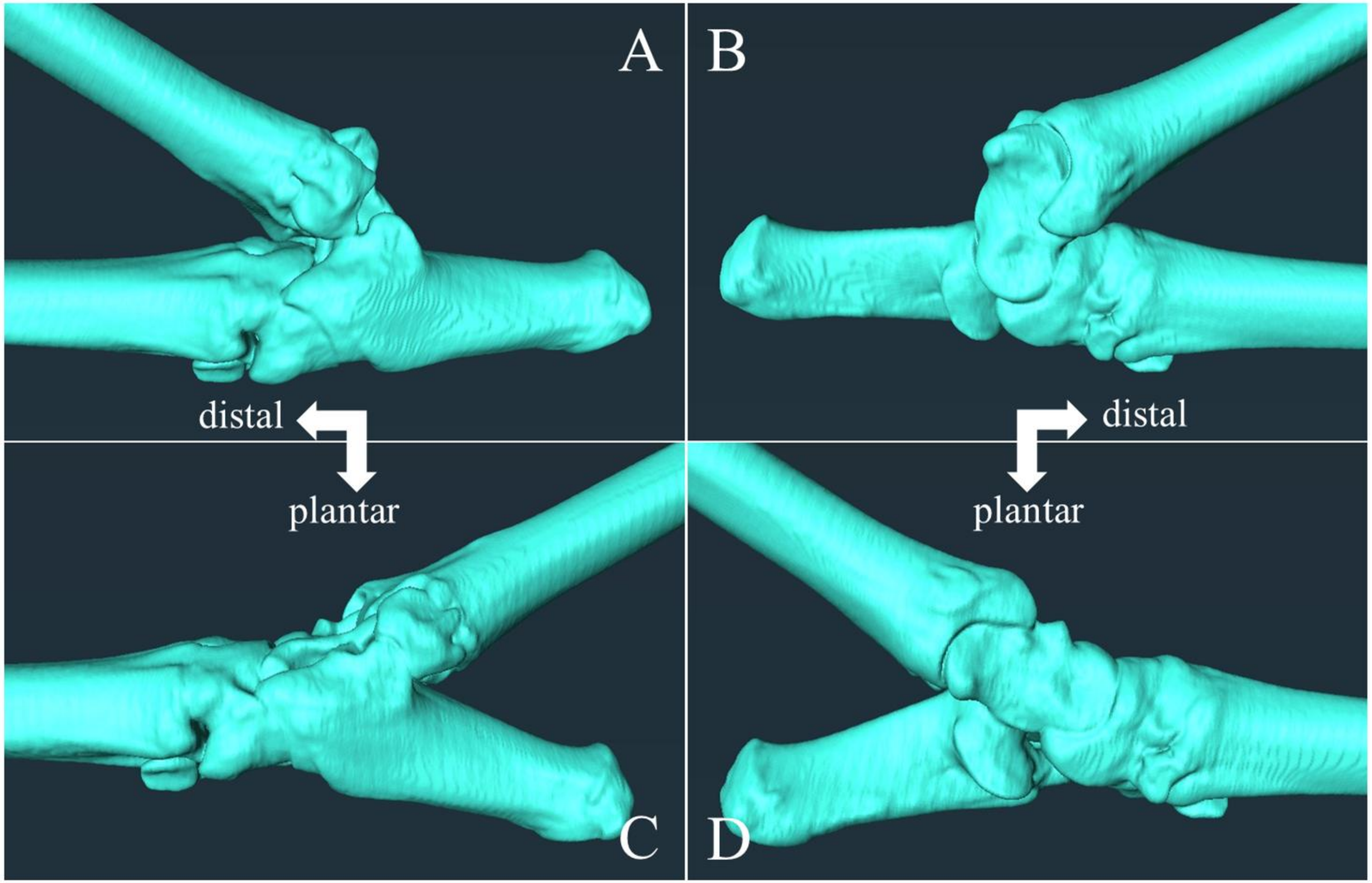

Ovis canadensis (UMUT-24422)

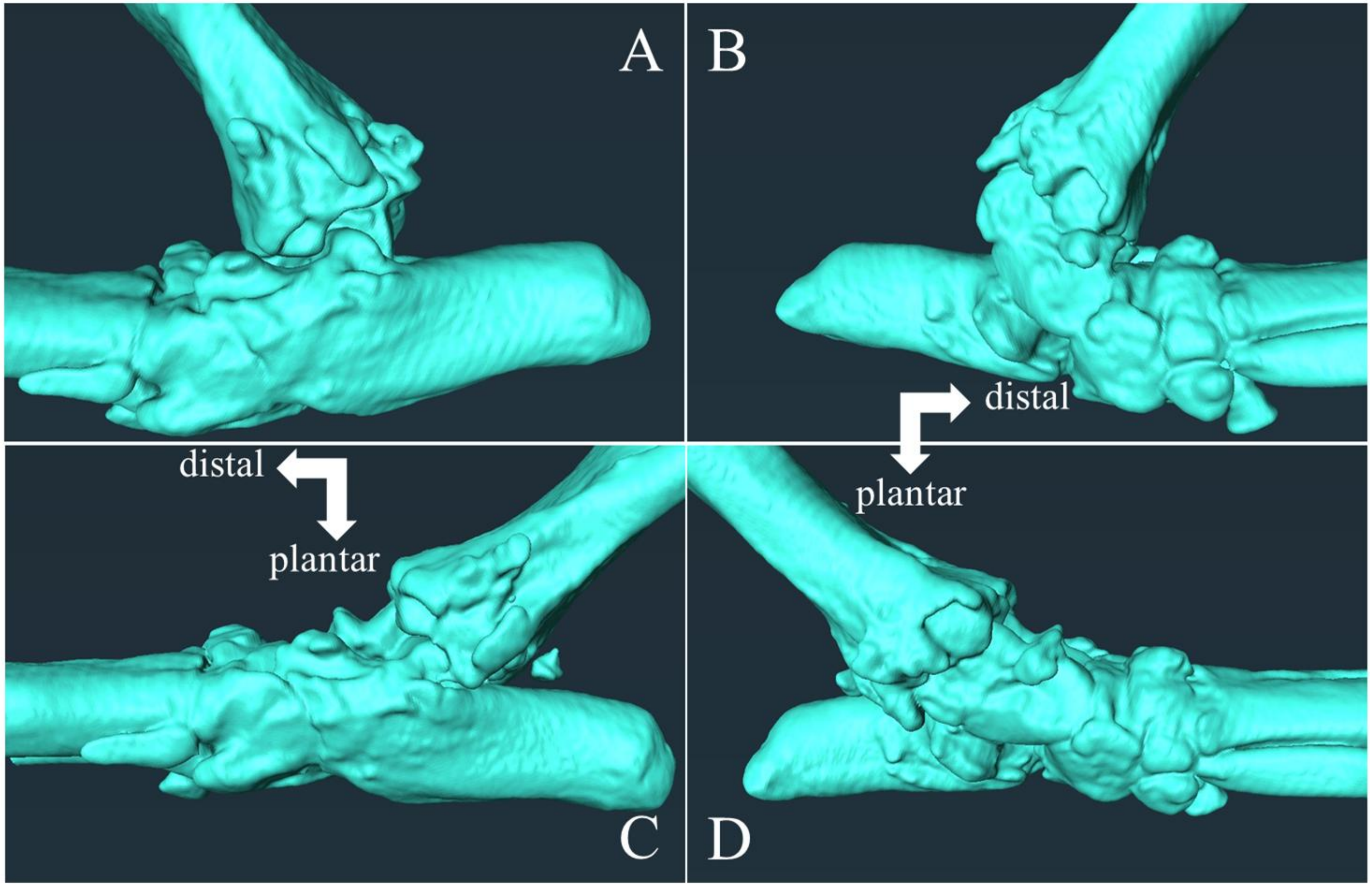

Pecari tajacu

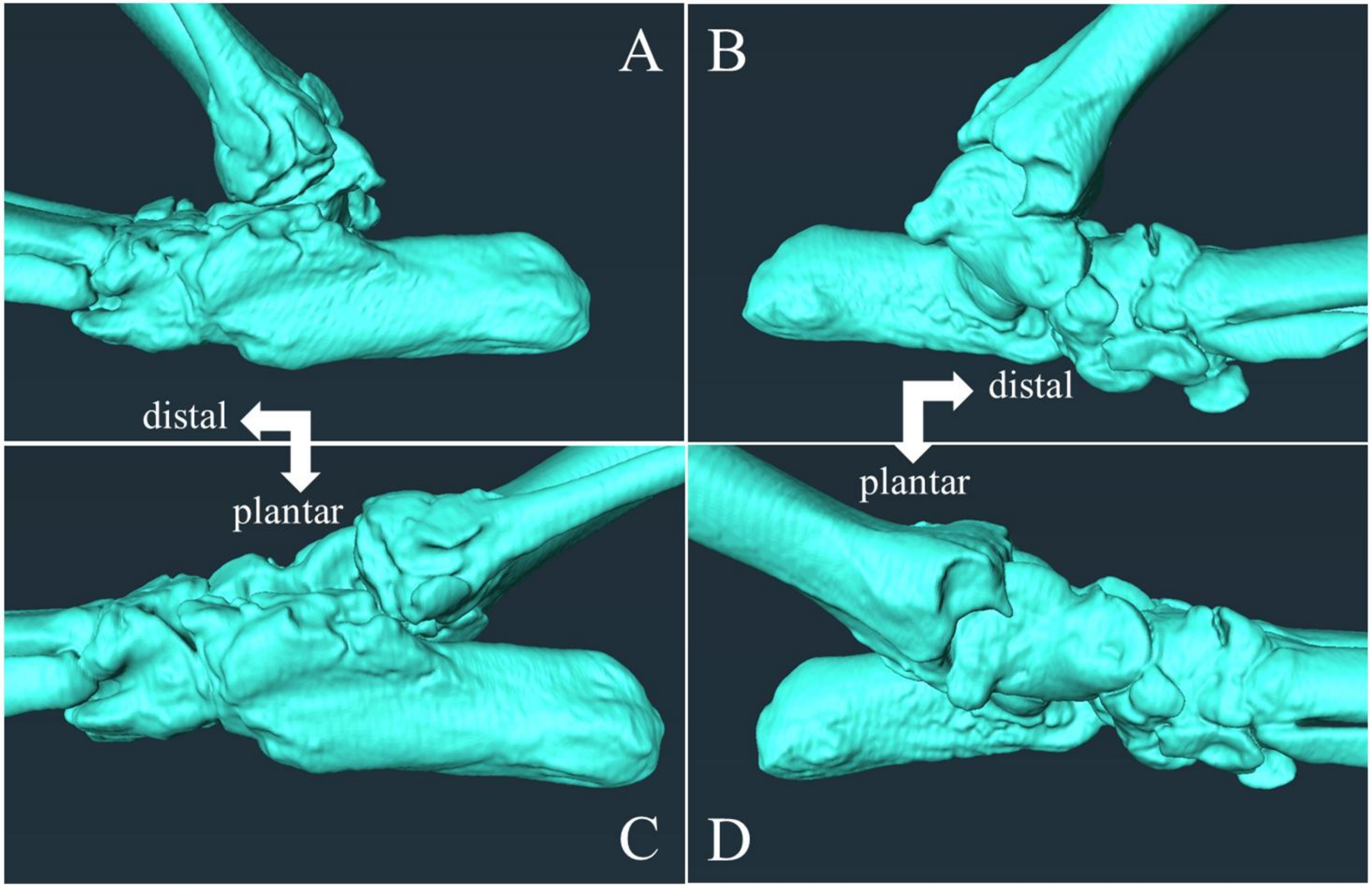

Potampchoerus porcus

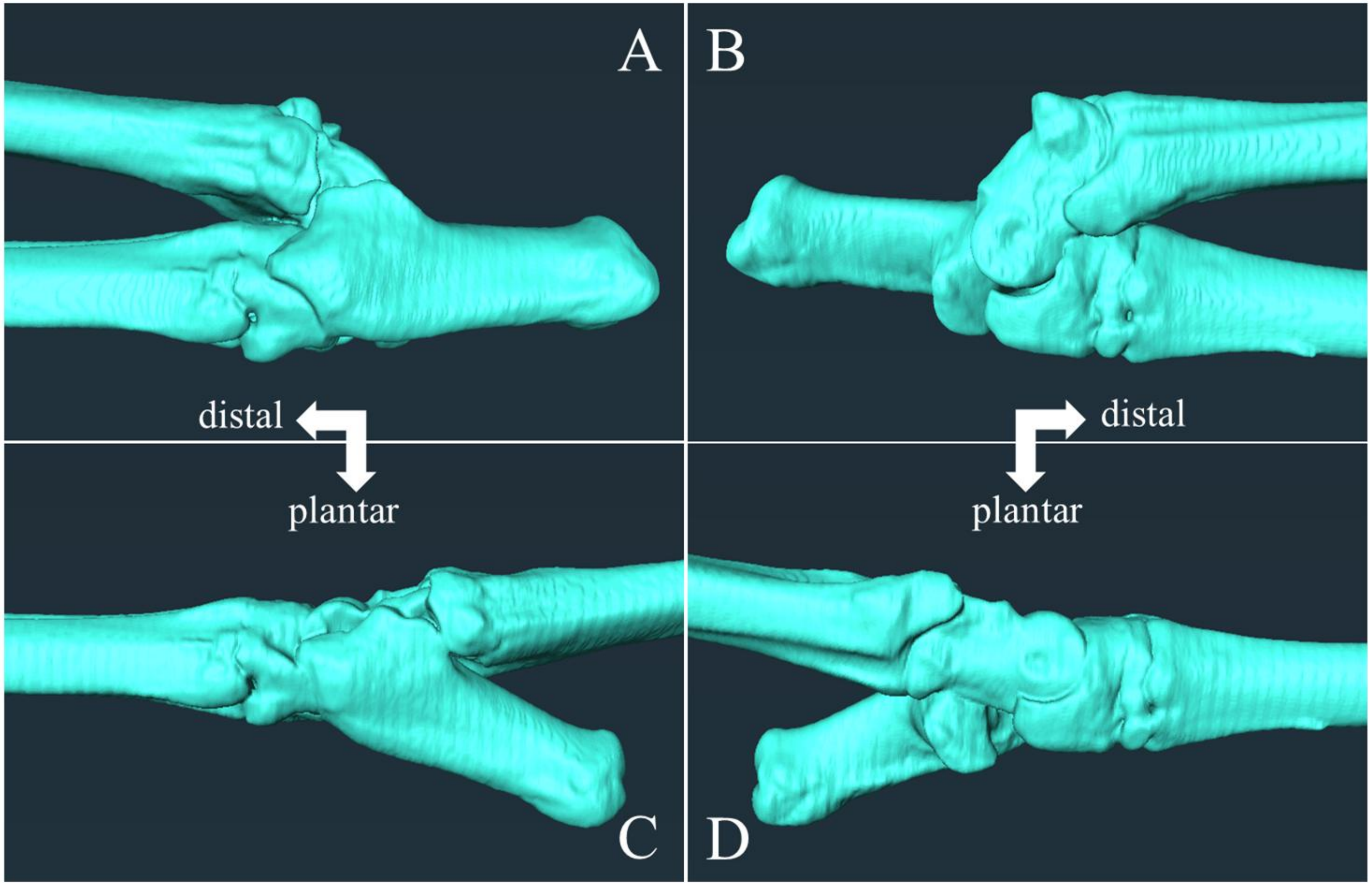

Tragelaphus spekii

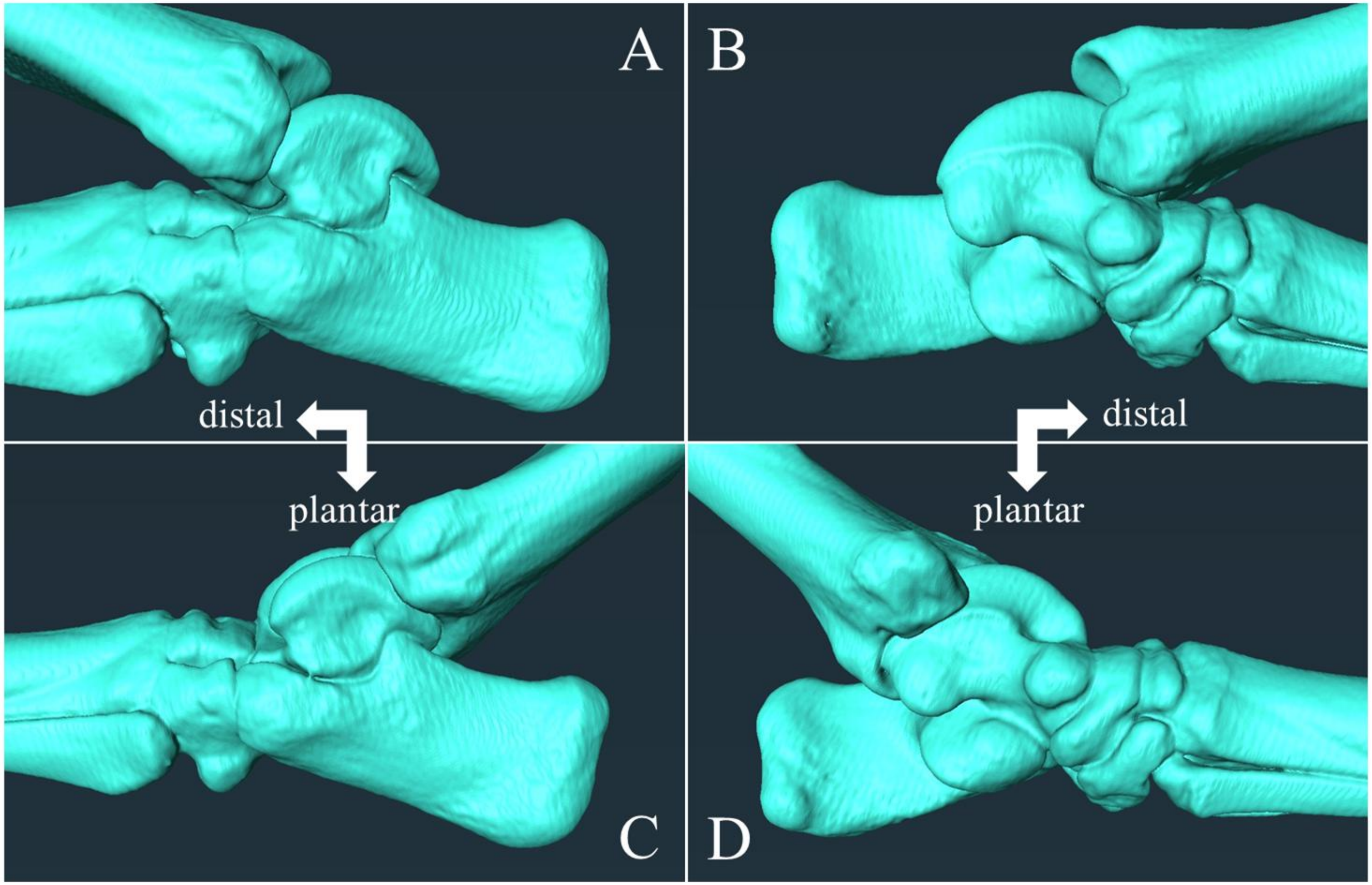

Equus asinus

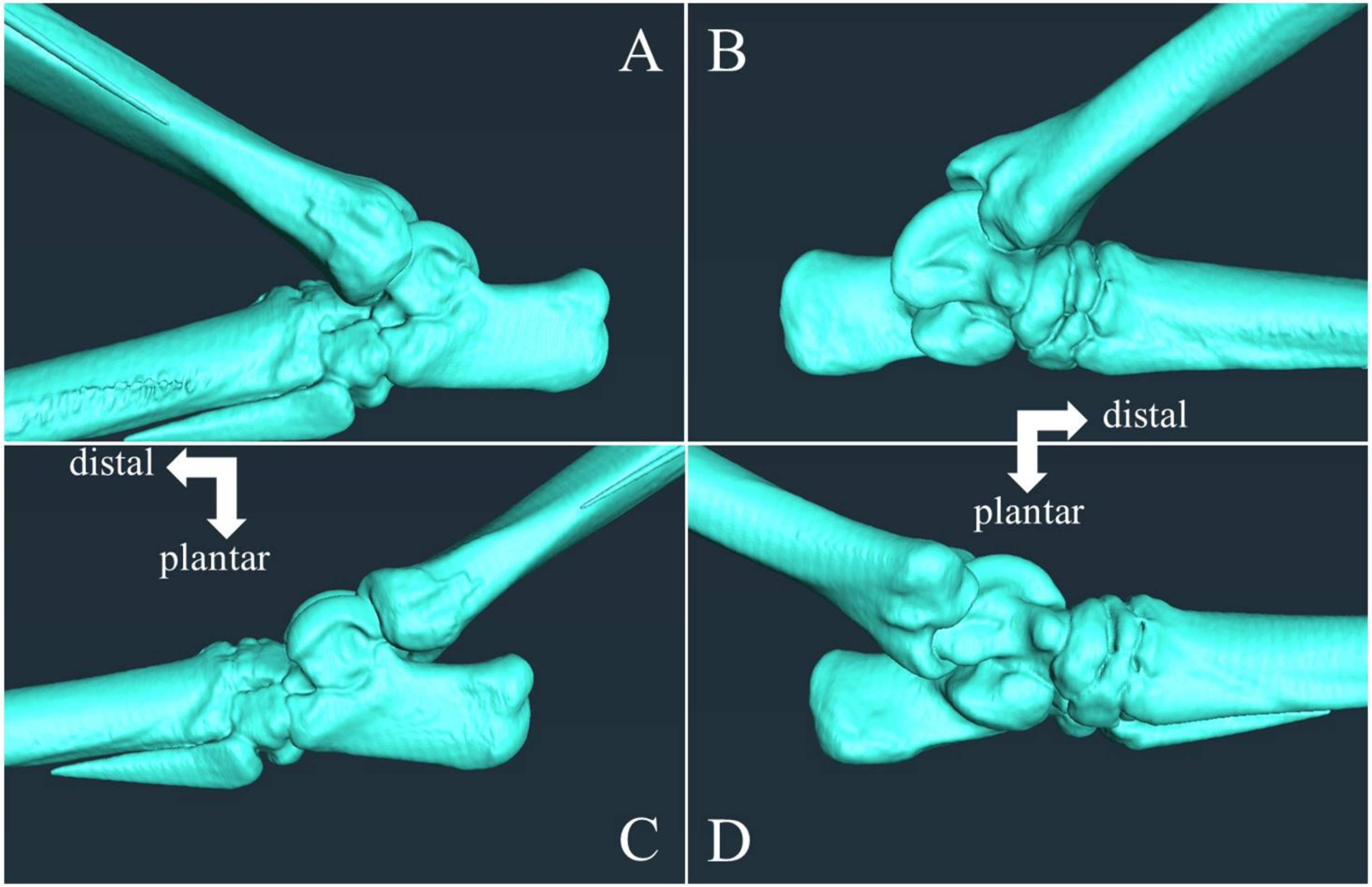

Equus caballus

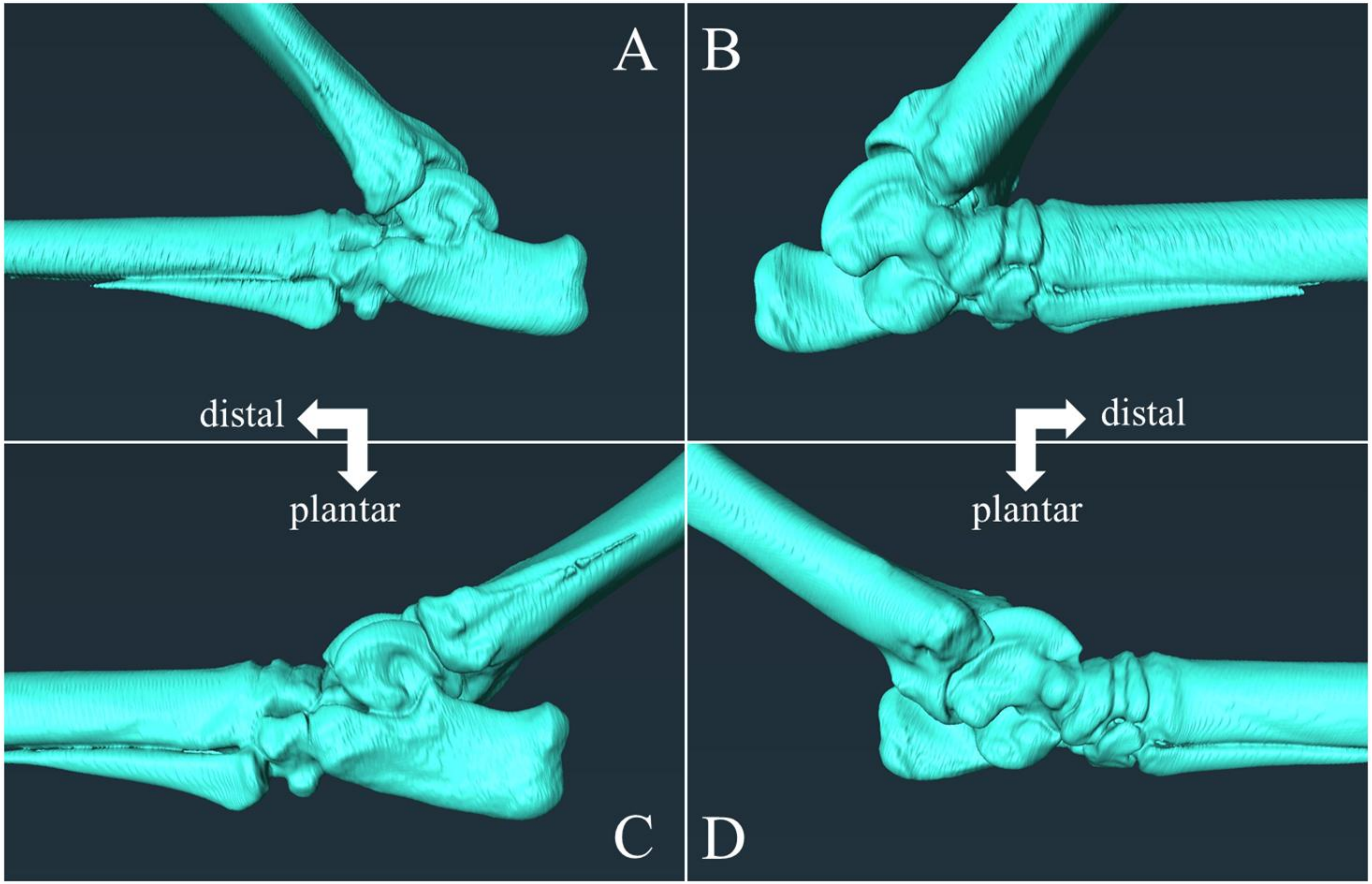

Equus grevyi

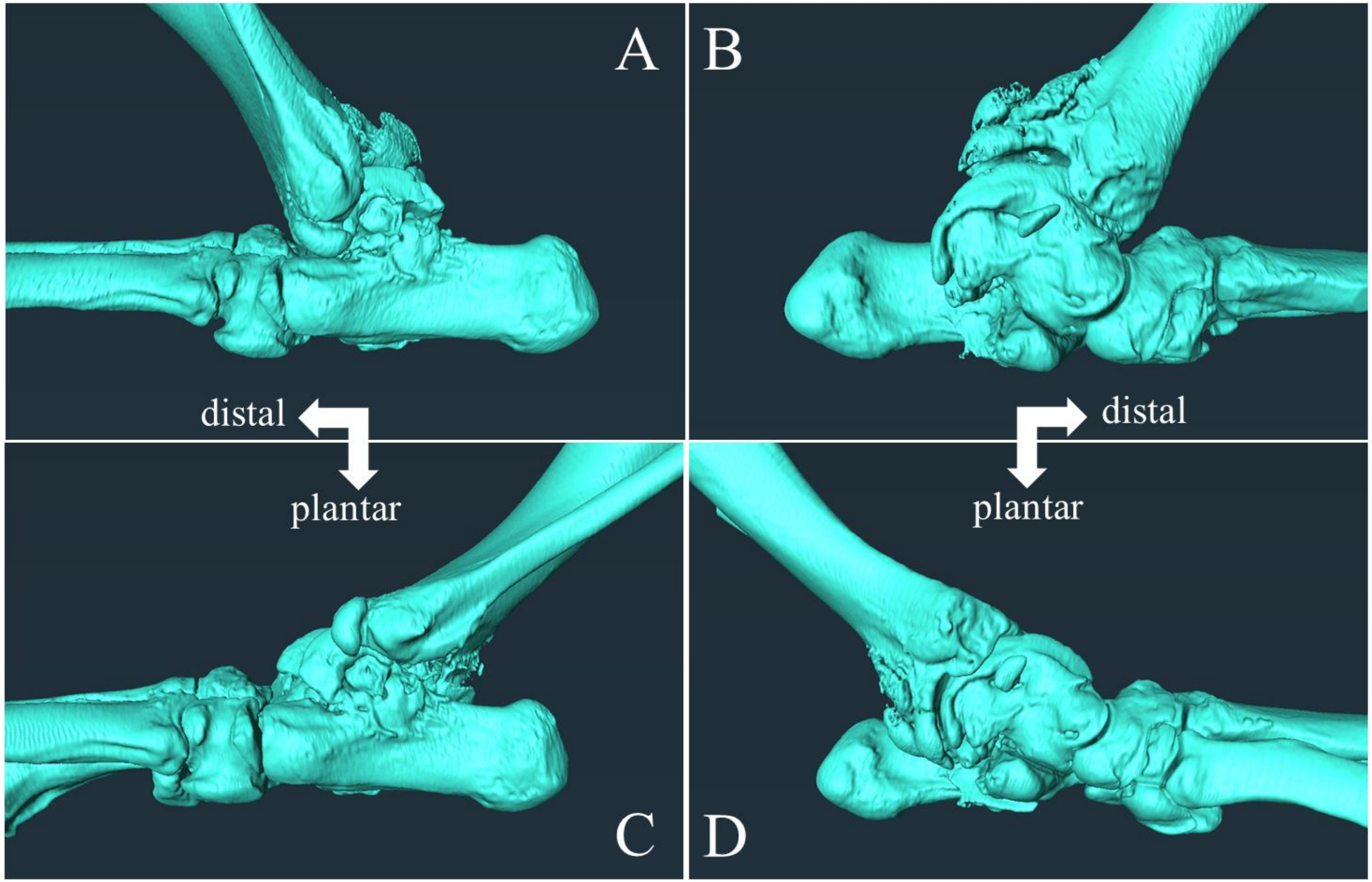

Tapirus indicus (UMUT-23030)

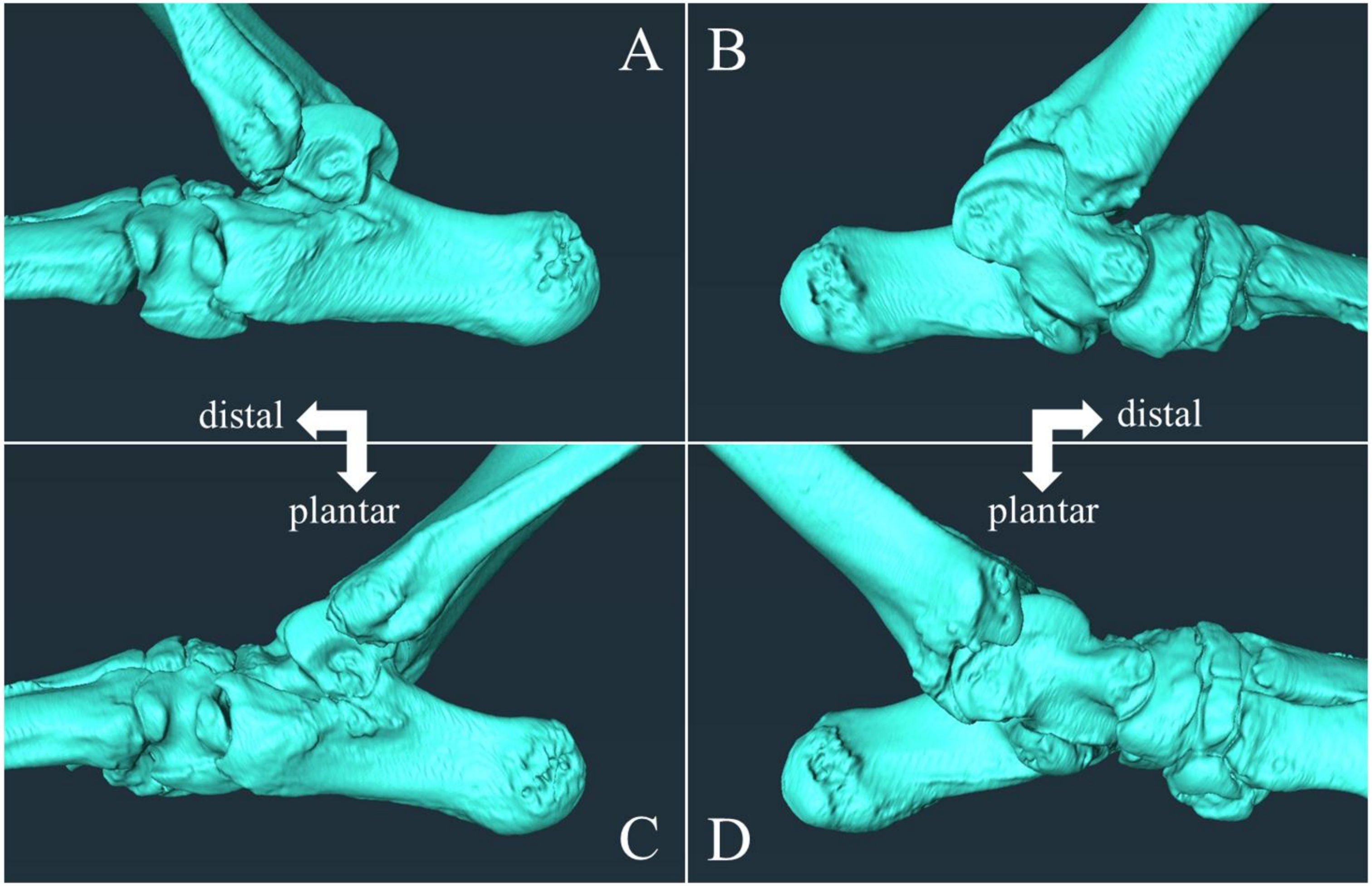

Tapirus indicus (UMUT-24201)

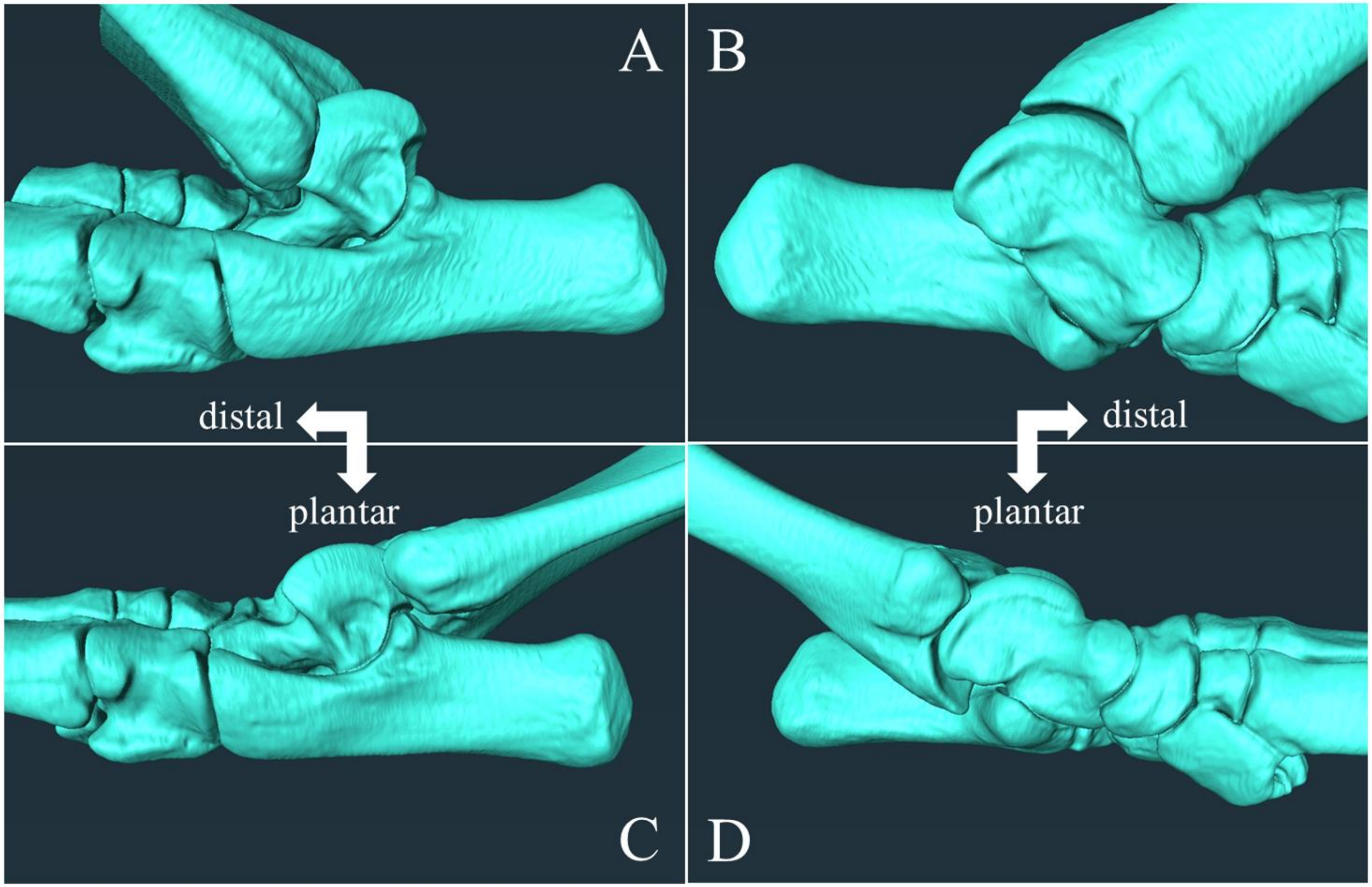

Tapirus terrestris

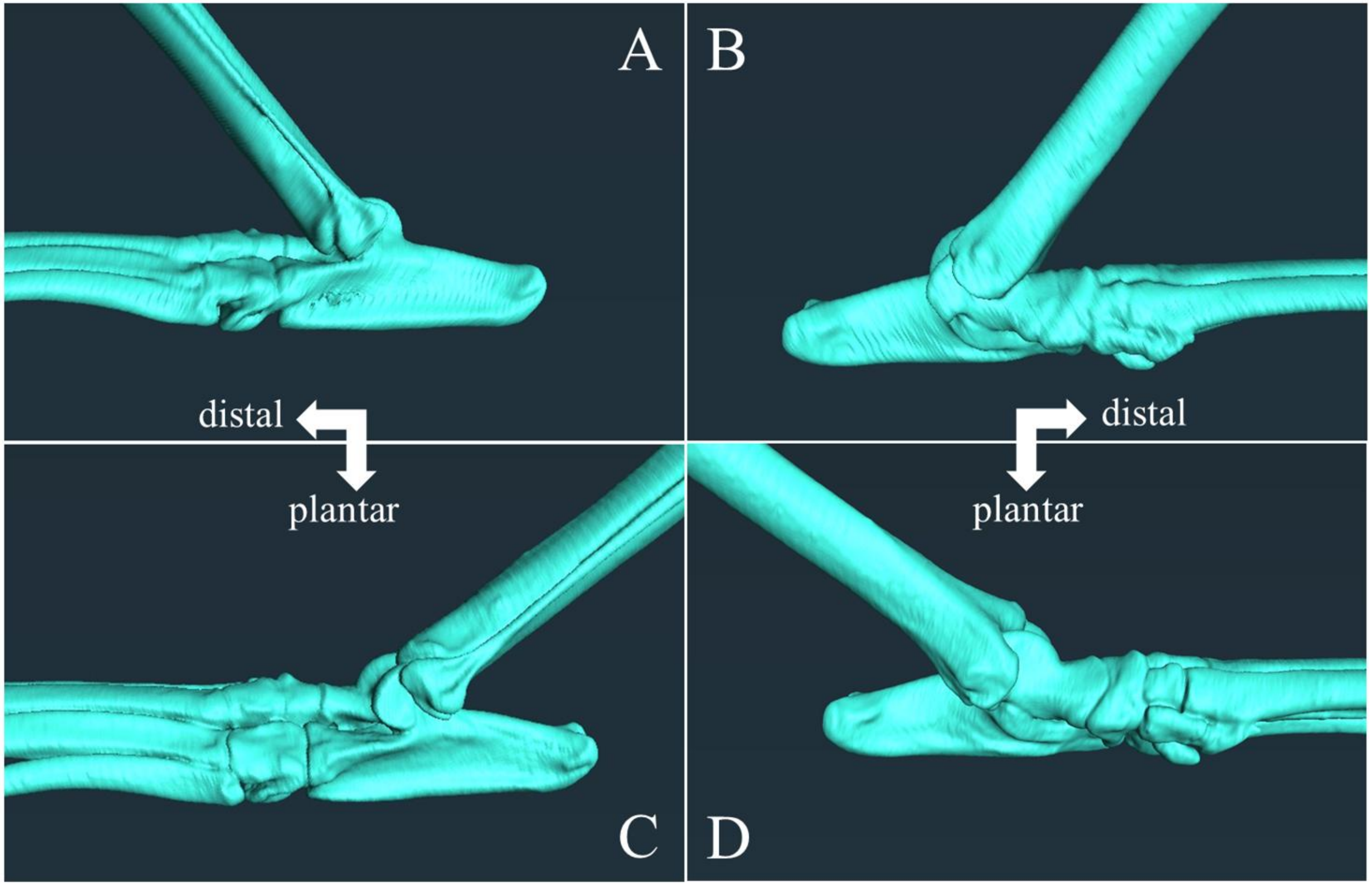

Acinonyx jubatus
